# A set of powerful negative selection systems for unmodified Enterobacteriaceae

**DOI:** 10.1093/nar/gkv248

**Published:** 2015-03-23

**Authors:** Varnica Khetrapal, Kurosh Mehershahi, Shazmina Rafee, Siyi Chen, Chiew Ling Lim, Swaine L. Chen

**Affiliations:** 1 National University of Singapore, Department of Medicine, Yong Loo Lin School of Medicine, 1E Kent Ridge Road, NUHS Tower Block, Level 10, Singapore 119074; 2 Genome Institute of Singapore, Infectious Diseases Group, 60 Biopolis Street, Genome, #02-01, Singapore 138672

## Abstract

Creation of defined genetic mutations is a powerful method for dissecting mechanisms of bacterial disease; however, many genetic tools are only developed for laboratory strains. We have designed a modular and general negative selection strategy based on inducible toxins that provides high selection stringency in clinical *Escherichia coli* and *Salmonella* isolates. No strain- or species-specific optimization is needed, yet this system achieves better selection stringency than all previously reported negative selection systems usable in unmodified *E. coli* strains. The high stringency enables use of negative instead of positive selection in phage-mediated generalized transduction and also allows transfer of alleles between arbitrary strains of *E. coli* without requiring phage. The modular design should also allow further extension to other bacteria. This negative selection system thus overcomes disadvantages of existing systems, enabling definitive genetic experiments in both lab and clinical isolates of *E. coli* and other Enterobacteriaceae.

## INTRODUCTION

Elucidation of the molecular basis for a given phenotype in bacteria (such as the ability to cause an infection) is heavily reliant on the ability to create defined genetic mutations. Numerous systems exist for manipulation of bacterial chromosomes; in general, the most powerful of these use selection to isolate the desired mutant. Positive selection markers based on antibiotic resistance genes are thus a mainstay of the genetic toolbox in nearly every genetically tractable bacterium.

**Figure 1.**
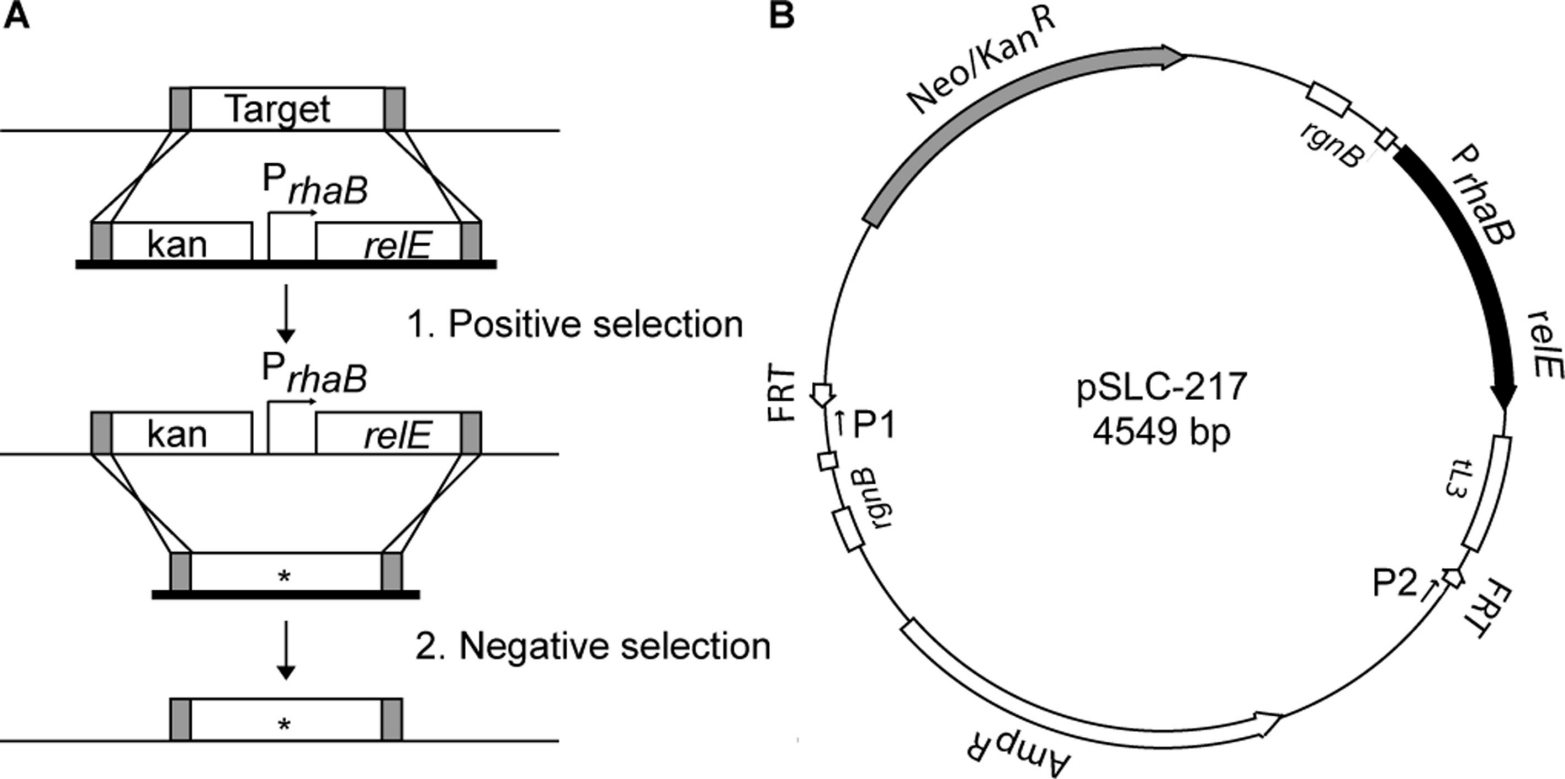
Design and characterization of a negative selection module. (**A**) Schematic of a two-step cloning strategy for allelic replacement using positive and negative selection. *Step 1*. A PCR product (depicted with a thick black baseline) containing a positive–negative selection cassette (kan-P*_rhaB_-relE* here) flanked by sequence (shaded gray) homologous to the targeted gene is subjected to double crossover recombination (indicated by crossing lines) to replace the gene. Selection for this replacement is done using the positive selection marker (kanamycin here). *Step 2*. Another PCR product (thick black baseline) containing the same or a different allele (indicated by *) of the target gene, also flanked by sequence homologous to the targeted locus is subjected to double crossover recombination to reintegrate the original locus. The resulting genome carries no residual markers or DNA scar. Selection for this reintegration is done using the negative selection marker (plating on rhamnose in this case). (**B**) Schematic diagram of template plasmid pSLC-217. The negative selection module (P*_rhaB_* driving *relE*) is shaded in black. The positive selection module (*neo*, mediating kanamycin resistance) is shaded gray. Transcriptional terminators are indicated by open boxes. Universal priming sites P1 and P2 are shown as arrows. Other features (FRT sites, Amp^R^ gene) are indicated by open box arrows.

However, cloning strategies relying solely on positive selection markers (which enable isolation of bacteria carrying the marker) result in ‘marked’ strains, where the marker itself or a residual scar remains in the genome, potentially causing unanticipated effects. Creation of definitive genetic constructs (e.g. a single point mutation in a gene of interest) is therefore greatly facilitated by negative selection markers (which allow selection of bacteria without the marker). Having negative selection enables a two-step strategy of (i) positive selection-mediated deletion/replacement of a gene of interest by a selection cassette followed by (ii) negative selection-mediated, seamless replacement of the same cassette by a mutated allele (Figure 1A). Comparison with another strain in which the selection cassette is similarly replaced with a wild-type allele thus allows rigorous assignment of phenotypic differences to the engineered point mutation.

Negative selection markers are in general less efficient than positive selection markers, even for well-studied bacteria such as *Escherichia coli* (1–8). Particularly for disease-causing clinical isolates, negative selection systems that require host genotype modification (e.g. *ccdB, tolC, thyA, rpsl* and thymidine kinases (4–8)) are impractical, as the required host modifications often involve conserved metabolic functions that may impact virulence (9), thereby confounding the analysis or requiring an additional step to restore the original genotype. Among negative selection systems that are usable in unmodified host strains (and are therefore candidates for direct use in clinical isolates), additional drawbacks include low selection stringency (as for *sacB* (2)) and the need for strain-specific optimization of selection conditions (as for the *tetA* and *tetA–sacB* systems (1,3)).

To overcome these disadvantages in unmodified hosts, we designed a general and modular negative selection system based on inducible toxins. We optimized selection conditions in one clinical strain of *E. coli*, UTI89, then verified that this design was usable without further modification or optimization in lab and other clinical isolates of *E. coli* as well as in *Salmonella enterica*, enabling convenient two-step creation of markerless and scarless chromosomal point mutations. The negative selection module achieved high selection stringency in all tested strains, exceeding all other reported negative selection systems usable in unmodified lab isolates of *E. coli* by up to 60-fold, and approaching theoretical predictions of maximum stringency based on mutation rates. The high negative selection stringency enabled its use in traditional phage-mediated generalized transduction, in which an additional benefit is the generation of unmarked transductants using a wild-type donor. Furthermore, we were able to use the negative selection system to perform a modified phage-free ‘transduction’ (which we term generalized allelic exchange) using transformed whole genomic DNA between two uropathogenic isolates of *E. coli*, an otherwise difficult problem due to the lack of transducing phages for clinical strains (10). Therefore, this negative selection system is a convenient and powerful addition to the genetic toolbox for all *E. coli* strains that, in combination with traditional antibiotic markers, enables the creation of arbitrary unmarked mutations in all tested strains of *E. coli* and other Enterobacteriaceae and allows for definitive genetic experiments even in clinical strains. It further enables new applications for generalized allelic exchange between different strains.

## MATERIALS AND METHODS

### Media and culture conditions

LB and M9 agar were used as complex and minimal media, respectively. For plasmid maintenance and selection of positive selection antibiotic markers, media was supplemented with antibiotics (Sigma, Singapore) at the following concentrations: ampicillin (100 μg/ml), kanamycin (50 μg/ml), chloramphenicol (20 μg/ml) and tetracycline (10 μg/ml). M9 agar was supplemented with 0.2% glucose to repress toxin gene expression or with 5% sucrose (for *cat-sacB* selection), 0.2% rhamnose (for all P*_rhaB_*-driven toxins) or 0.2% arabinose (for P*_araB_*-driven toxins). Maintenance of plasmids carrying the toxin genes was carried out in LB supplemented with 2% glucose.

### Bacterial strains and plasmids

Relevant characteristics of bacterial strains and plasmids used in this study are listed in Supplementary Table S1. The template plasmids pKD3 and pKD4 were obtained from AddGene and were modified in accordance with the AddGene Material Transfer Agreement for non-commercial purposes.

### *tetA* negative selection

Overnight cultures of UTI89 carrying *tetA* on plasmids or integrated into the chromosome were diluted 1:100 into fresh media and grown to an optical density at 600nm (OD_600_) of 1. Serial 10-fold dilutions were plated to quantify the number of surviving bacteria (CFU) on selective and non-selective (LB) plates. Selective plates were supplemented with tetracycline (for positive selection) or 2–4 mM NiCl_2_ (for negative selection).

### Plasmid construction

All plasmids used in this study are listed in Supplementary Table S1. To facilitate cloning, we first inserted several restriction sites (AatII, SmaI and SacII) into the AfeI site of pKD4, generating pSLC-243. Toxic genes were amplified from *E.coli* MG1655 (*relE, chpBK, mqsR, higB, yafQ* and *yhaV), Pseudomonas aeruginose* (*tse2* (11)) or *E. coli* UTI89 (phage λ holin (12)) genomic DNA by PCR then cloned using NdeI and BamHI sites (in pAH120) or NdeI and NheI (in pAH150 (13)) to place them under the control of the *rhaB* or *araB* promoter, respectively. Each promoter-toxin module, including the flanking tL3 and *rgnB* transcription terminators from pAH120 or pAH150, was cloned by blunt ligation into the SmaI site of pSLC-243 to generate template plasmids (pSLC-17 containing kan-P*_rhaB_-relE* is shown in Figure 1B). All plasmids were propagated in *E. coli* strain BW23473 (*pir*+, low copy number) or BW23474 (*pir116*, high copy number). The sequences of all negative selection cassettes are available upon request.

### Recombineering

All genomic manipulations were carried out using previously described Red recombinase recombineering protocols optimized for lab or clinical isolates of *E. coli* (14), with minor modifications. The positive–negative selection cassette was amplified by PCR using the P1 and P2 universal primers for pKD4. Primers used for cloning are listed in Supplementary Table S8. Primers used for *fimH* constructs were as previously described (15). Each primer was synthesized with an extra 50 bp of sequence at the 5′ end that was homologous to the targeted genomic locus. Red recombinase was expressed from the pKM208 (in *E. coli*) or pKD46 (in *S. enterica* Serovar Typhimurium 14028S) helper plasmids. Overnight cultures (LB-ampicillin, 30°C with agitation) of the target strain were diluted 1:100 into fresh media, grown at 30°C with agitation to OD_600_ = 0.2–0.3, and induced with 1 mM Isopropyl β-D-1-thiogalactopyranoside (IPTG) (pKM208) or 0.2% arabinose (pKD46) for 30–45 min. The induced cells were then heat shocked for 15 min at 42°C, followed by swirling in an ice water bath for 15 min. Cells were harvested by centrifugation (5000 rpm for 10 min), washed two times with cold (4°C) water, re-suspended in 1/100 of the original culture volume of cold 10% glycerol, and frozen in 50 μl aliquots as competent cells. To perform double crossover homologous recombination, 1 μg of a PCR product was transformed by electroporation into thawed competent cells using 1mm electroporation cuvettes in a GenePulser XCEL set to an output voltage of 1500 V with 25 μF capacitance and 400 Ω resistance (Bio-Rad, Singapore). Cells were then recovered in LB at 37°C with shaking for 2 h followed by static incubation (no shaking) for 2 h, plated on selective plates, and incubated overnight (for kanamycin selection) or for 24–48 h (for rhamnose selection) at 37°C.

### Diagnostic PCR and Sanger sequencing

Colony PCR reactions were used to check for insertion/replacement of the selection cassette. The first primer pair was used to specifically detect the locus of desired homologous recombination and the second primer pair was to detect the presence or absence of the specific toxin gene at the desired locus. Primers used are listed in Supplementary Table S8. Primers for *fimH* constructs were uti8 + 4913333 and uti8 − 4915222 as described previously (15). The strains were confirmed using Sanger sequencing of the diagnostic PCR products with the same primers used for amplification (1st Base, Singapore).

### Immunoblot

Bacteria from 1 ml of culture at OD_600_ = 1.0 was harvested by centrifugation (14 000 rpm, 1 min). The bacterial pellet was resuspended in 300 μl of 4× sodium dodecyl sulfate (SDS) loading buffer (50% glycerol, 0.5% SDS, 0.25 M Tris, mercaptoethanol and bromophenol blue). Ten microliters of this sample was analyzed by sodium dodecyl sulphate-polyacrylamide gel electrophoresis (SDS-PAGE) (15% polyacrylamide gel). Proteins were transferred to a nitrocellulose membrane using a Trans-Blot^®^ SD Semi-Dry transfer cell (Bio-Rad, Singapore). Blots were probed with anti-GroEL antibody (Sigma, Singapore) at a 1:6000 dilution in Tris-Borate-EDTA (TBE) with 5% skimmed milk to assess loading quantities. Blots were probed with custom anti-OmpC monoclonal antibodies (Abmart Inc., Shanghai, China). Antibody 4H15 (1:1000 dilution) recognizes both the wild type and mutant OmpC protein (mutated in loop 5 residues) while 4K3 (1:1000) was raised against the OmpC loop 5 residues and thus does not detect the mutant OmpC protein. Secondary antibodies used were ECL™ anti-rabbit IgG Horseradish peroxidase (HRP)-linked whole antibody (1:10 000) for anti-GroEL antibody and ECL™ anti-mouse IgG HRP-linked whole antibody (1:10 000). Blots were visualized using Amersham HRP substrate in a Chemidoc machine (Bio-Rad, Singapore).

### Whole genome sequencing

Genomic DNA from strain SLC-561 (UTI89 *fimH*::FRT; *mlaA*::*mlaA*^UTI89^) was prepared from 1mL of an OD_600_ of 1 culture using the Wizard^®^ Genomic DNA Extraction Kit (Promega) and prepared for Illumina sequencing using the TruSeq DNA library preparation kit (Illumina) according to the manufacturer's recommendations. This library was sequenced on a HiSeq 2000 (2 × 76 bp reads), yielding 5 680 917 read pairs (∼170× coverage). After quality filtering, fastq files were analyzed with cortex_var (version 1.0.5.20) from the Cortex package (16) using a joint analysis with UTI89 (NCBI accession number NC_007946) as the reference genome; the resulting VCF files indicated the known deletion at the *fimH* locus but no other variants (insertions, deletions, or single nucleotide polymorphisms) that passed quality filters. The sample was further tested for large (>100 bp) structural variations relative to the UTI89 reference genome using the SVRE program (Sukumaran and Chen, unpublished data) ; no large structural variations besides the *fimH* deletion were found that differed from wt UTI89.

### Hemagglutination titers

These were performed as previously described (15). Briefly, 1 ml of an OD_600_ = 1.0 suspension of bacterial cells in PBS was gently pelleted (4000 × g, 2 min) and resuspended in 100 μl phosphate buffered saline (PBS). Twenty-five microliters of this was serially diluted in a row of a 96-well V-bottom plate (Corning #3897) where each well contained 25 μl of PBS (with or without mannose) (dilution range 1:2 to 1:4096). Twenty-five microliters of guinea pig red blood cells in PBS were added to each well. The plate was gently agitated and incubated overnight at 4°C. The HA titer reported was the greatest dilution of cells that resulted in visible clumping of erythrocytes.

### SDS–EDTA sensitivity

Overnight cultures were diluted 1:100 into fresh media and grown to OD_600_ = 1. Serial 10-fold dilutions were plated on different media to quantify the number of surviving bacteria (CFU). Selective plates were supplemented with 0.5% SDS and between 0.75–2.25 mM ethylenediaminetetraacetic acid (EDTA) and non-selective plates were supplemented with 0.5% SDS, with or without kanamycin.

### Selection stringency

Overnight grown cultures were diluted 1:100 into fresh media and grown to OD_600_ = 1. Serial 10-fold dilutions were carried out and plated on non-restrictive (plain LB for all strains; also including antibiotic plates for antibiotic resistant strains) media to quantify the number of surviving bacteria (CFU) in the original suspension. 10^10^ cells (10 ml culture of OD_600_ = 1, centrifuged and re-suspended with 200 μl PBS) were spread on appropriate restrictive plates to check for negative selection (on M9-rhamnose or M9-arabinose for strains carrying the negative selection cassette) and positive selection (LB-kanamycin for strains not carrying the kanamycin positive selection marker) stringency. Selection stringency was determined by dividing the calculated CFU of the original inoculum on restrictive plates by the average number of CFU that grew on non-restrictive plates.

### Luria–Delbruck fluctuation test

Strains were grown overnight on a non-restrictive plates (LB for MG1655 and LB-kanamycin for SLC-568) and one single colony was resuspended in 50 μl of PBS. Twenty microliters of this suspension was diluted 200× and aliquoted into the central 60 wells of each of 2× 96-well plates per strain. Each well received 20 μl of the diluted suspension (approximately 10^2^ or 10^4^ CFU/well) and 180 μl of non-restrictive media (LB for MG1655 and M9 with 0.2% glucose for SLC-568). The plates were then incubated at 37°C with shaking (220 rpm) for 6 h (MG1655, starter cultures with 10^4^ CFU/well) or 18 h (SLC-568, starter cultures with 10^2^ CFU/well). (As a control, we also carried out 8-h incubations with SLC-568; the resulting data was similar to that from experiments using an18-h incubation). Ten wells from each plate were used to calculate total CFU per well by serial dilutions. The remaining 50 wells from each of the two plates were pelleted (100 total) and resuspended in 200 μl of selective medium (LB-kanamycin for MG1655 and M9 with 0.2% rhamnose for SLC-568). The plates were then incubated overnight at 37°C with shaking (220rpm) after which the cells were again pelleted and resuspended in 10 μl of restrictive medium and spotted on restrictive agar plates. The agar plates were incubated at 37°C for 24–48 h. The number of wells which gave rise to colonies growing on restrictive agar plates was counted and the mutation rate of the strains was determined using the following formula (17);

\begin{eqnarray*} &&{\rm Mutation}\,{\rm rate} = \nonumber \\ &&-\ln \frac{{({\rm no}.\,{\rm of}\,{\rm negative}\,{\rm samples}/{\rm total}\,{\rm no}.\,{\rm of}\,{\rm samples})}}{{{\rm total}\,{\rm no}.\,{\rm of}\,{\rm cell}\,{\rm divisions}}} \end{eqnarray*}

### Generalized transduction

Transduction using P1*vir* was performed in MG1655 using standard protocols (18). A transducing lysate generated from wild type MG1655 was used to infect SLC-568 (MG1655 *hsdS*::kan-P*_rhaB_-tse2*) followed by plating on M9-rhamnose as described above in the ‘Recombineering’ section.

### Generalized allelic exchange

Isolated genomic DNA from *E. coli* CFT073 was sheared to an average length of 10 kb fragments by sonication (Branson Digital Sonifier Disruptor 450) (2 × 5 s pulses at 10% power on ice). Ten micrograms of sheared DNA was transformed into competent SLC-557 (UTI89 *ompC*::kan-P*_rhaB_-relE*) expressing red recombinase. Cells were recovered in LB at 37°C with shaking for 2 h followed by static incubation (no shaking) for 2 h then plated on M9-rhamnose agar plates, and screened via PCR and sequencing for allelic exchange at *ompC* locus using P21 and P22 primer set.

### BLAST analysis of type II toxins

Type II toxin genes present in MG1655 were taken from (19) and from the NCBI RefSeq annotation of the MG1655 genome (NC_000913). Finished *E. coli* genome sequences were downloaded from NCBI RefSeq (Supplementary Figure S2). Each non-K12 genome was used as the database to search for each toxin gene using the blastn (using the toxin gene sequences) and tblastn (using the toxin protein sequences) programs (BLAST 2.2.28+) with default parameters. Examination of the blastn and tblastn results showed that a cutoff of a blastn alignment over 80% of the toxin gene length captured all genes with simultaneously >88% DNA identity and >84% protein identity over at least 80% of the toxin gene (or protein) sequence. This 80% blastn alignment cutoff also included four additional gene/genome combinations which had potential frameshift mutations (*yafQ* and *yafO* in SMS_3_5; cbtA in O55_H7 RM12579 and P12b) but for which the entire gene sequence was otherwise present. We thus used 80% alignment over the toxin gene length by blastn as the cutoff to call toxins as present or absent in a given genome.

## RESULTS

### Existing negative selection systems are not practical for a clinical isolate of *E. coli*

We first tested those existing negative selection systems that do not require host genotype modification for their ability to function in single copy in the chromosome of an unmodified clinical uropathogenic isolate of *E. coli*, UTI89. Only two systems, *tetA* (1) and *sacB* (20), could potentially be used in UTI89. Selection against Tet resistance by fusaric acid is known to require optimization in different strains of *E. coli* and may not be usable in others (21); we were unable to demonstrate negative selection mediated by the *tetA* gene found on pBR322 in UTI89 after optimization of fusaric acid and salt concentrations (data not shown). It has also been reported that Ni^2+^ salts can be used to select against Tet^R^ strains and that the selectivity can in some cases exceed 1 in 10^6^ cells (1), but the selection power and optimal Ni^2+^ concentration varied between different *E. coli* strains, typically requiring above 6mM NiCl_2_. We found that, indeed, growth of UTI89/pBR322 (Tet^R^) relative to UTI89 was inhibited by 4 orders of magnitude at an optimal concentration of 3 mM NiCl_2_. However, pBR322 has an estimated copy number of 20/cell. In order to use this system for chromosomal manipulation, it must work at a copy number of 1/cell. We found that the sensitivity of Tet^R^ UTI89 to NiCl_2_ decreased with copy number, to the point that no NiCl_2_ sensitivity was found with UTI89::tet (containing a single copy chromosomal *tetA* gene) relative to UTI89 (Supplementary Figure S1), as was also seen with DH10B (a lab adapted strain of *E. coli*) (1).

Gram negative bacteria carrying the *B. subtilis sacB* gene accumulate toxic metabolites when grown in the presence of sucrose. The *sacB* gene is widely used for negative selection (20,22,23), but it suffers from relatively high false positive rates for chromosomal manipulations due to spontaneous suppressor mutations that inactivate *sacB* (24). This has the overall effect of reducing its stringency to <2.3 × 10^−5^ (20). However, in UTI89, a single chromosomal copy of *cat-sacB* mediated negative selection with a relatively weak selection stringency of 3.40 × 10^−2^. Recently, a system combining both the *tetA* and *sacB* genes has been reported with improved stringency (3) but is very sensitive to selection conditions and therefore requires strain-specific optimization. Therefore, a negative selection system immediately usable in clinical strains of *E. coli* is lacking.

### Design of a modular negative selection cassette based on inducible toxins

We sought to design a new negative selection system that would both provide high stringency and not require strain-specific optimization. Negative selection using the *mazF* toxin gene has been demonstrated in single strains of *Bacillus subtilis* (25) and *Clostridium* *acetobutylicum* (26), though no data on stringency was reported. These reports suggested that isolated toxins from toxin–antitoxin (TA) systems could be generally useful as negative selection markers, even when used in heterologous species (*mazF* is an *E. coli* gene, yet seems to function in Gram positives). We therefore asked whether a general system providing tightly controlled induction of a toxin gene could be used in *E. coli* and other Enterobacteriaceae. The *E. coli* MG1655 (a lab-adapted fecal isolate) chromosome is known to carry numerous TA systems (19); we focused on Type II TA systems (in which both toxin and antitoxin are proteins) because they are more easily identified and therefore likely to be more completely annotated. To develop a system for use in clinical isolates (in particular, UTI89), we reasoned that the TA system we used should be absent from the UTI89 genome. Using BLAST analysis, we identified 12 candidate toxins present in MG1655 but not in the UTI89 genome (Supplementary Table S2). Further analysis using 55 non-K12 full genome sequences available at NCBI RefSeq highlighted 7 MG1655 toxins (*rnlA, ypjF, ykfI, ydaS, yjhX, relE* and *mqsR*) that were not found in more than half of non-K12 genomes (Supplementary Figure S2 and Supplementary Table S2).

We created a negative selection cassette consisting of the *relE* toxin (chosen for its small size, to minimize the PCR length in subsequent steps and its potential for inactivation by mutation) driven by the *rhaB* promoter (chosen for its ‘tight’ control; it is induced by growth on rhamnose and repressed by growth on glucose). To further ensure deliberate control of expression, we included the *tL3* and *rgnB* terminators flanking the P*_rhaB_-relE* to guard against read-through transcription from other promoters; and we retained the native *rhaB* ribosome binding site. For use in making unmarked chromosomal mutations, we combined this negative selection cassette with a kanamycin resistance gene, a traditional positively selectable antibiotic marker. We built the construct as a derivative of the well-designed cloning system described by Datsenko and Wanner (14) based on Lambda Red recombinase and antibiotic selection using the pKD4 template plasmid. We refer to the derived template plasmid as pSLC-217 and to the combined positive–negative selection cassette as kan-P*_rhaB_-relE* (Figure 1B).

### Efficient, scarless and markerless manipulation of the UTI89 chromosome

To validate our system, we first used the kan-P*_rhaB_-relE* cassette to perform a two-step allelic replacement of the *ompC* gene at its native locus in UTI89. We used a Red recombinase protocol optimized for clinical isolates of *E. coli* (22) to replace the native *ompC* gene by homologous recombination with a linear PCR product (containing kan-P*_rhaB_-relE* flanked by homology arms targeting the *ompC* locus), using positive selection on kanamycin (Figure 1A, step 1). We then used PCR products for the wild type UTI89 *ompC* and a mutated *ompC* (carrying mutations in extracellular loop L5) allele in a subsequent Red recombinase-mediated double-crossover replacement of the integrated positive–negative selection cassette, using negative selection on rhamnose (Figure 1A, step 2). Clones were screened using PCR for the entire *ompC* locus as well as for specific insertion of the *relE* toxin gene at the *ompC* locus (Supplementary Figure S3A). Positive clones identified by PCR were verified by testing for loss of kanamycin resistance, restored ability to grow on rhamnose (Supplementary Figure S3B), and Sanger sequencing of the entire *ompC* locus. The efficiency for isolating the correct recombinant during the counterselection step was 87–100% as calculated by testing 30 colonies from each counterselection step by PCR, growth on rhamnose, and loss of the positive selection marker. To further confirm that the restored *ompC* locus was functional, we tested for OmpC protein expression using immunoblotting. We saw OmpC protein expression in whole cell extracts for the parental wild type strain as well as both recombined strains (carrying a wt and L5 mutant *ompC*, respectively), but no OmpC band in the intermediate knockout strain. Furthermore, using an antibody specific to the L5 loop of OmpC, we saw detection only in the parental wild type (wt) and the strain with the restored wt *ompC* allele but not in the knockout or the strain carrying the L5 mutant allele (Supplementary Figure S3C). Therefore, the combination of our negative selection cassette with a traditional positive selectable marker has enabled us to create a functional, scarless mutation directly in the *ompC* locus of UTI89.

### Creation of markerless mutations at arbitrary loci in UTI89

We verified that the kan-P*_rhaB_-relE* positive–negative selection cassette could be used to manipulate arbitrary genes in UTI89 by performing gene deletion and complementation at three other loci (Supplementary Table S3). We used the two-step integration/knockout followed by replacement of the integrated dual selection cassette, using negative selection on rhamnose to create isogenic mutant strains or unmarked mutations with defined deletion junctions (Supplementary Tables S1 and S3). We successfully deleted and reintegrated the *mlaA* and *hsdS* genes, verifying each by both diagnostic PCR and Sanger sequencing of the locus. Functional confirmation of *mlaA* knockout and reintegrated constructs was also done using an SDS–EDTA sensitivity assay (Supplementary Table S4). We also made unmarked versions of previously reported strains carrying point mutations in the *fimH* gene (15), eliminating the downstream linked kanamycin gene previously required for the second step of cloning. All clones were confirmed to contain the predicted mutations by diagnostic PCR and sequencing of the new junctions created by recombination. Furthermore, all of the unmarked *fimH* mutants were verified to have similar type 1 pilus function to their marked counterparts based on hemagglutination of guinea pig red blood cells (Supplementary Table S5).

### No off-target mutations are created

Expression of Red recombinase can induce a 10-fold increase in spontaneous mutations (22). As the goal is to perform well-defined chromosomal manipulations, the two rounds of Red recombinase mediated recombination are a potentially large problem for off-target, unintended mutations. To mitigate this, we followed the recommendations of Campellone and Murphy to minimize Red recombinase induction to 30–45 min prior to generation of competent cells in both recombination steps (22). To further address whether off-target mutations were being generated, however, we performed whole genome sequencing on a strain that had undergone a chromosomal knockout then reintegration of the *mlaA* gene. Using Cortex (which detects SNPs and small indels) (16), we found no mutations compared with the parental strain (prior to the initial knockout of *mlaA*). We also used a new sensitive structural variation caller (which detects larger inversions, duplications, insertions, deletions and other more complex rearrangements; manuscript in preparation) and again found no differences in genomic rearrangements compared with the parental strain. While we only examined one strain, the complete absence of any detectable off-target mutations in over 5 Mbp of sequence in this strain indicates that unintended mutations are not generally being created at a high rate during these engineering steps.

### Generalization of negative selection for other host strains by module replacement

The positive–negative selection cassette we created was built from several well known genetic elements (promoters, terminators, RBS sequences and antibiotic markers). In the interest of generality, potentially enabling use in other *E. coli* strains as well as non-*E. coli* bacteria, we first verified that we could simply replace the promoter and antibiotic marker without having to perform any optimization of sequences or selection conditions. We created P*_araB_-relE*, where the negative selection would be carried out with growth on arabinose. We also switched the parental template plasmid to pKD3, encoding a chloramphenicol positive selection cassette (Supplementary Table S3). Indeed, switching between P*_rhaB_* and P*_araB_* or chloramphenicol and kanamycin had little effect on the efficiency of the negative selection step (based on limited testing of 7–8 colonies by PCR and loss of the positive selection marker) when used in a similar two-step allelic replacement at the *ompC* locus of UTI89 using the previously defined selection conditions we used for UTI89 (although the antibiotic and sugar were altered as necessary).

Given that several toxins encoded by MG1655 besides *relE* were also potential candidates for use in UTI89, but that may be variably present in other *E. coli* strains, we next tested whether we could also simply replace the toxin gene in a modular fashion. We tested several other toxins (*mqsR, higB, yhaV, yafQ* and *chpBK*) with P*_rhaB_*, using the same cloning sites we used for *relE* to make identical fusions of the start codon of each toxin gene with the promoter and ribosome binding site of our template plasmid. We further reasoned that alternative toxins, such as those from phage or antibacterial secretion systems, might function effectively both in *E. coli* (thus enabling use in MG1655 and other lab strains) as well as in other bacteria. We therefore also included the holin gene (324 bp) from phage λ (12) in UTI89 and the *tse2* type 6 secretion system toxin (477 bp) from *Pseudomonas aeruginosa* (11). These positive–negative selection cassette variants were again tested using the same two-step knockout/replacement protocol without modification of transformation or growth conditions. We found that, except for variants containing *higB* and *yafQ* (see below), all variants were usable for creating markerless mutations in UTI89.

Finally, we tested our system in different bacterial strains. We replaced the *ompC* gene in CFT073 (another uropathogenic *E. coli* clinical isolate (27)), EDL933 (an enterohemorrhagic *E. coli* isolate (28)), TOP2515 (a cystitis isolate that has never been reported to be manipulated on its chromosome (29)), and *S. enterica* serovar Typhimurium 14028S (30) with the *ompC* allele from UTI89 (Supplementary Table S3). In addition, we replaced the *hsdS* gene in *E. coli* MG1655 with the *hsdS* gene from UTI89 (Supplementary Table S3) using the *P. aeruginosa tse2* gene as the negative selection system toxin. All replacements were successful as verified by PCR, growth phenotypes, and Sanger sequencing. Furthermore, for all variant cassettes and all strains tested, we used the same amplification primers, transformation conditions, and negative selection conditions (M9 with 0.2% rhamnose) that we had originally used for UTI89. Thus, our positive–negative selection cassette is indeed general and needs no optimization when used in different clinical isolates of *E. coli* and other Enteric bacteria.

### Inducible toxins mediate high negative selection stringency

**Figure 2.**
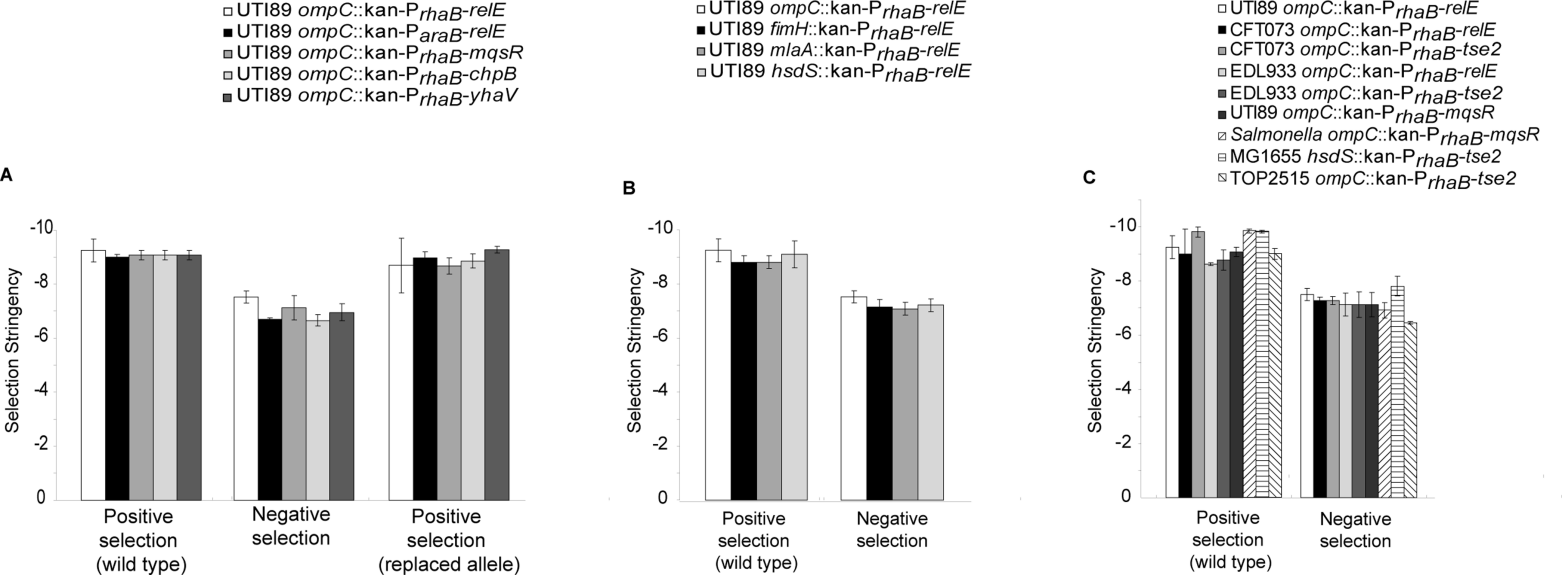
Selection stringency tests. (**A**) Selection stringency tests for different toxins. Data shows the selection stringency, which is calculated as: CFU/ml on restrictive agar divided by CFU/ml on non-restrictive agar. First set of bars represents the positive selection stringency for wild type (UTI89) strains tested in each experiment. The middle set of bars represents the negative selection stringency for each strain carrying the dual selection cassette integrated into the *ompC* locus in UTI89. The last set of bars represents positive selection stringency for the strains in which the selection cassette has been replaced with the wild type *ompC* allele in a subsequent negative selection step. (**B**) Stringency does not vary with chromosomal locus. Data shows the positive selection stringency for wild-type strains (on the left) followed by the negative selection stringency for strains carrying the kan-P*_rhaB_-relE* cassette at different loci in UTI89 (on the right). (**C**) The positive–negative selection cassette is usable in different strains of *E. coli* and in *Salmonella*. Toxins and strains are as indicated. For graphs in (A–C), data represents the average of the log-transformed values of selection stringencies with error bars indicating standard deviation of the log-transformed stringency values calculated from three independent biological replicates.

We noted in these manipulations that, in general, very few background/false positive colonies grew on rhamnose plates (Supplementary Figure S3B). Furthermore, two toxins, *higB* and phage λ holin, could suppress growth on rhamnose when present on a high copy plasmid but not when present in single copy on the chromosome (Supplementary Figure S4). These data suggested that different toxins vary in selection stringency, which might be useful for creating negative selection cassettes in organisms lacking tightly regulated promoters. We first tested selection stringency by calculating the fraction of viable colony forming units (CFUs) that were able to grow under restrictive conditions (smaller numbers indicate higher stringency). This test measures the frequency of colonies growing under restrictive conditions (as opposed to the actual mutation rate – see below), is naturally related to the background colonies one would observe during a typical cloning procedure, and has been used previously in rapid screens of mutability (31–33). We therefore refer to these values as ‘stringency frequencies’. We plated bacteria carrying a positive–negative selection cassette in single copy in the chromosome onto each of LB, LB-antibiotic and M9-rhamnose or M9-arabinose plates. On non-restrictive plates, we quantified bacteria by plating serial dilutions of the original culture, while on restrictive plates, we concentrated 10^10^ CFU from 10 ml of OD_600_ = 1 culture and plated them all. For reference, the parental strains and the strains with the replaced alleles (both sensitive to kanamycin or chloramphenicol) were also plated to the same plates to test the stringency of positive selection on antibiotics (Figure 2A). We found that positive selection markers mediated very strong selection (stringency frequencies of 10^−9^–10^−10^) in UTI89 (Figure 2A). Among negative selection markers, different toxins, when driven by the P*_rhaB_* promoter, did indeed vary in their strength of selection, but the toxins that were usable for negative selection in single copy on the UTI89 chromosome had stringency frequencies of 10^−7^–10^−8^ (Figure 2A and Supplementary Table S6). Because the insertion site of a negative selection cassette may affect its relative copy number due to the timing of DNA replication, and copy number influences expression levels and thereby potentially stringency, we repeated these tests with the kan-P*_rhaB_-relE* selection cassette integrated into different loci in UTI89. In general, stringency did not vary much across different loci within UTI89 (Figure 2B, Supplementary Figure S5A), across different *E. coli* strains, or in *Salmonella* (Figure 2C, Supplementary Figure S5B). Notably, the *tse2* toxin (11) from the *Pseudomonas* Type VI secretion system had the highest stringency frequency (1.96 × 10^−8^) at the *hsdS* locus in MG1655 (Figure 2C). In comparison, the highest stringency for any negative selection system reported to date for unmodified *E. coli* strains (MG1655 or its close relatives, all lab-adapted) is 6 × 10^−7^ (presumably also a stringency frequency) using a combined *tetA*–*sacB* cassette (3).

We verified that CFU quantification on rhamnose plates was accurate by comparing CFU calculated by serial dilutions plated to both plain LB and M9-rhamnose; no differences were seen for any of the wild-type strains (Supplementary Table S7). Furthermore, we also tested a control in which the kanamycin gene and P*_rhaB_* were integrated into the chromosome without an accompanying toxin gene (SLC-658); this also showed no toxicity from growth on rhamnose compared with plain LB (Supplementary Table S7).

To further validate the high stringency of our system, we explored several additional lines of reasoning for the strain carrying the *tse2* toxin in MG1655 (SLC-568). First, we repeated our initial stringency frequency test by plating a higher number (10^11^ CFU; total culture volume 100 ml, OD_600_ = 1) of cells onto M9-rhamnose plates; we found a similar stringency frequency (1.11 × 10^−8^; Supplementary Figure S6), representing growth of at least several hundred colonies in each of three replicates of this experiment.

Second, we estimated the theoretical maximum possible stringency (i.e. lowest mutation rate) based on chromosomal replication error rates for MG1655, assuming that only mutations within the negative selection cassette itself could confer breakthrough growth. The range of mutation rates for MG1655 has been reported to be between 1 × 10^−3^ and 3.3 × 10^−3^ /genome/generation (34,35); this translates into 2.2 × 10^−10^ to 7.1 × 10^−10^ mutations/nucleotide/generation. The P*_rhaB_-tse2* cassette is 811 bp; therefore the expected mutation rate is 1.8 × 10^−7^ to 5.8 × 10*^−7^* mutations/cassette/generation. Not all mutations would inactivate the toxin or disrupt its expression; this fraction is difficult to quantify, but we take as limits the expected fraction of coding mutations that would be expected to be nonsense (5%) (36) and the fraction of *lacI* mutations that are dominant (40%) (37) (this range encompasses the estimated fraction (20%) of missense mutations (∼70% of all *de novo* mutations) that are strongly deleterious (overall 14% of all *de novo* mutations)) (36). This gives an expected inactivation rate between 0.9 × 10^−8^ and 2.3×10^−7^/cassette/generation. In addition the toxin could be inactivated by a spontaneous large deletion, which occurs at a rate of between 1.79 × 10^−9^ and 2.5 × 10^−7^ per generation in *E. coli* (38–40). These calculated mutation rate values are now *rates* at which colonies growing on rhamnose could be generated, and should be considered distinct from the stringency frequencies measured and discussed above.

Measurement of experimental *rates* of mutation (as opposed to stringency *frequencies*) is more involved; the relevant issues have been previously elucidated by Luria and Delbruck (17). We carried out a Luria–Delbruck fluctuation test to directly measure experimental *rates* of mutation to breakthrough growth on rhamnose. Using two different starting small culture sizes (see ‘Materials and Methods’ section), we calculated a mutation rate toward growth on rhamnose of 1.42 × 10^−8^, within the theoretical range based on per-nucleotide mutation rates calculated above. As a control, we also performed a Luria–Delbruck fluctuation test for mutations conferring resistance to kanamycin. The fluctuation test gave a mutation rate to resistance (1.11 × 10^−10^) that was lower than our rate for mutation of our negative selection cassette, in keeping with the better stringency frequency of kanamycin (2.30 × 10^−10^) as measured above.

As a final verification of the reproducibility of the high stringency of our negative selection cassettes, we performed the same cloning to create a separate but isogenic strain to SLC-568 (designated SLC-657) and assayed its frequency of breakthrough growth on rhamnose (by plating 10^10^ CFU), again arriving at 2.38 × 10^−8^ (Supplementary Figure S6). We therefore conclude that the negative selection stringency (mutation frequencies) of the *tse2* system in MG1655 is indeed better than previously published negative selection systems and that its mutation rate is very close to the theoretical minimum mutation rate expected for a system of this size.

### Negative selection by inducible toxins enables unmarked allelic exchange

We then asked whether high negative selection stringency could be leveraged to enable ‘difficult’ applications such as generalized transduction, where positive selection markers are traditionally required (though recently P1 transduction has been performed using a *tetA-sacB* negative selection cassette (3)). We performed generalized transduction with a phage P1 lysate (generated from unmodified wild-type MG1655) to complement an *hsdS* knockout (replaced with kan-P*_rhaB_-tse2*; host strain MG1655 (SLC-568)); selection on rhamnose resulted in growth of >2000 transductants (out of ∼10^9^ plated CFU), of which 16/16 (100%) were positive for restoration of the wild-type *hsdS* locus by PCR. In contrast, no colonies were observed when the P1 phage lysate itself was plated, and 200 colonies were observed on rhamnose when only SLC-568 (the recipient strain) cells were plated. The estimated efficiency of >90% compares favorably with the 56–63% efficiency reported using *tetA-sacB* (3), consistent with the higher stringency of our system. Transduction using negative selection improves on traditional generalized transduction because the resulting transductants contain no residual antibiotic marker or scar and because unmarked, unmodified strains can be used as the donor.

**Figure 3.**
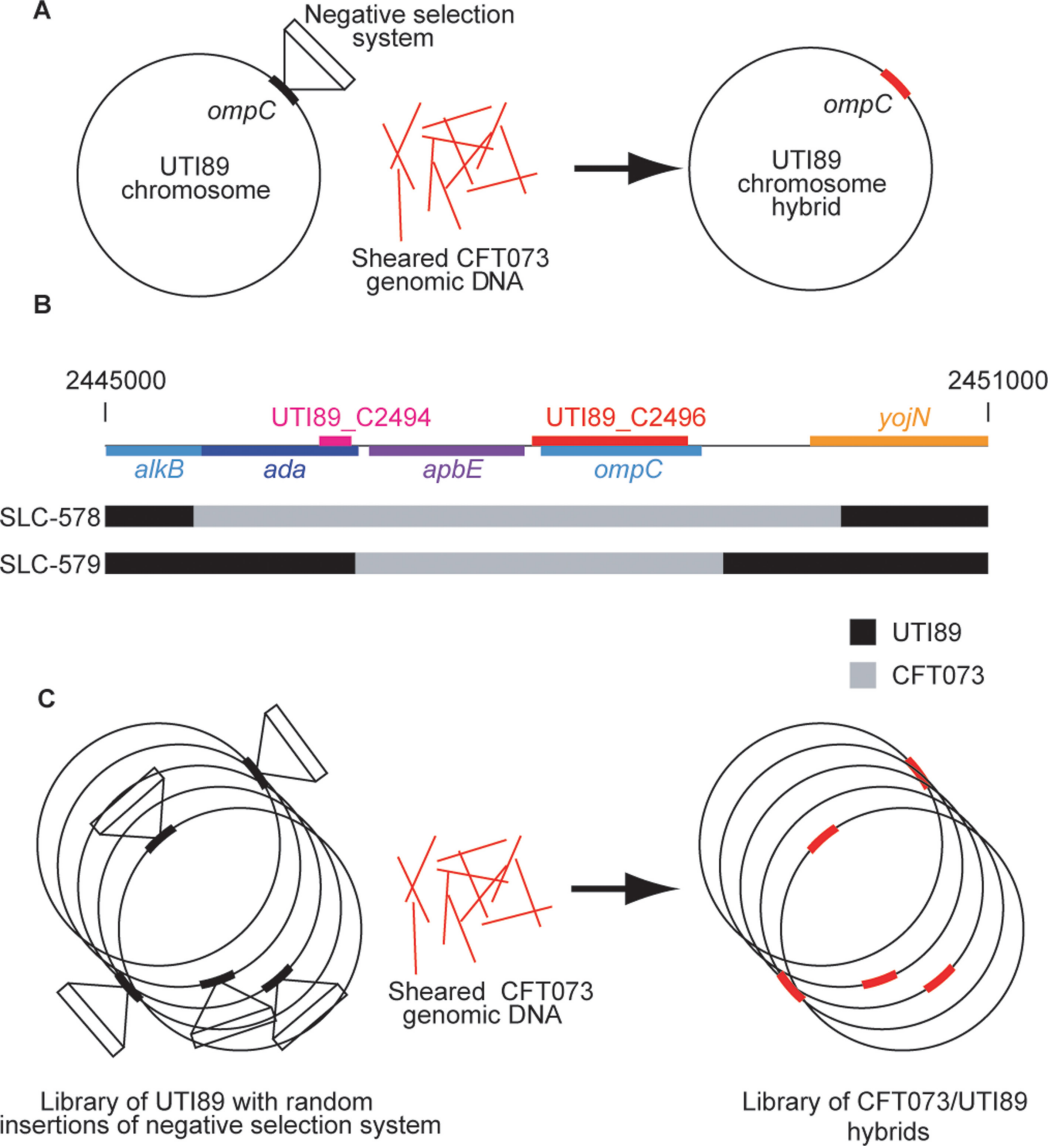
Generalized allelic exchange. (**A**) Schematic of the generalized allelic exchange strategy. A negative selection marker (open rectangle) is inserted into the recipient strain (UTI89 here) at a defined locus (thick black arc, *ompC* in this case). Sheared whole genomic DNA from another strain (CFT073 here, red lines) is transformed into UTI89, and growth on restrictive conditions allows selection for replacement of the targeted locus (thick red arc) with no residual marker or scar. (**B**) Recombination breakpoints for *ompC* allelic exchange from CFT073 to UTI89. A schematic of the gene organization of 6kb surrounding the *ompC* gene in UTI89 is shown at the top. The rectangles below depict recombination breakpoints of two clones (SLC-578 and SLC-579) as determined by Sanger sequencing of the *ompC* gene and flanking sequence. In each, a central region of CFT073 sequence encompassing the *ompC* gene is indicated in gray, while the surrounding UTI89 sequence is indicated by black. (**C**) Schematic for strain hybridization by mass generalized allelic exchange. A library of UTI89 with random insertions of the negative selection system (thick black arcs) throughout the chromosome is subjected to the process in (A) *en masse*. Growth on restrictive conditions would result in a library of hybrids in which each locus originally carrying the negative selection system insertion has been replaced by the homologous locus from CFT073 (thick red arcs).

However, clinical strains are often difficult to manipulate using generalized transduction, either due to phage specificity (10) or low efficiency. We were unable to perform a restoration of the *ompC* locus in UTI89 using P1-mediated generalized transduction from unmodified MG1655, and we were unable to create a P1 transducing lysate from either UTI89 or CFT073. We speculated that we could circumvent these difficulties by mimicking transduction using transformation of sheared genomic DNA (Figure 3A). Such a strategy would remove limitations imposed by the need to create a transducing lysate from the donor strain that could successfully infect the recipient strain. We refer to this phage-free ‘transduction’ as generalized allelic exchange. Reimplementation of generalized transduction in this way would not only expand its potential use to strains without transducing phage, but could also potentially lead to a novel application for strain hybridization we term ‘mass allelic exchange’ (see 'Discussion' section). We directly transformed sheared genomic DNA from CFT073 by electroporation into SLC-557 (UTI89 *ompC*::kan-P*_rhaB_-relE*) expressing Red recombinase. Selection on rhamnose followed by PCR screening resulted in identification of the desired recombinant, where CFT073 genomic DNA had replaced the *ompC* locus in UTI89, in 2/55 colonies (3.6%). We sequenced the DNA flanking *ompC* in these two clones and found that the precise recombination breakpoints were different (Figure 3B), as expected from the random shearing of the CFT073 genomic DNA. Of note, selection stringency is paramount in this application, as only 0.2% of the input DNA (the CFT073 genome is ∼5 Mbp, while DNA was sheared to 10 kb) is ‘on-target’ for replacement of the negative selection module at the *ompC* locus. Therefore, our negative selection system enables phage-free generalized allelic exchange between different strains of *E. coli*, substituting for generalized transduction.

## DISCUSSION

There is a surprising lack of stringent and general negative selection tools, even for cloning strains of *E. coli*. Indeed, several recent reports (3,4,41) have highlighted the need for better negative selection systems in *E. coli*. In general, previous systems have suffered from low stringency, a requirement for modified hosts, or a need to optimize selection conditions separately for each host strain. In the context of understanding the genetic basis of pathogenesis in primary clinical isolates, these drawbacks have made negative selection largely impractical. Particularly for loss of function studies, high frequency site-specific recombinase systems such as Cre-lox (42) and Flp-FRT (14) can be leveraged to manipulate chromosomal loci with only a small scar left behind, largely alleviating the need for negative selection. However, allelic replacement experiments in clinical isolates to date have been relatively rare compared with loss of function experiments; when done, they are limited either due to laborious construction methods or due to use of a linked positive selection marker.

General and stringent negative selection systems could remove these limitations on direct genetic studies of unmodified clinical pathogenic isolates. Recent reports of new negative selection systems have continued to focus on cloning strains of *E. coli* (3,4,41). These reported systems may be applicable to clinical strains, though the requirement for or complexity of strain-specific optimization is still unclear. Our system, due to its modular design, can accommodate essentially any toxin gene, whether from a toxin–antitoxin system, phage system, secretion system or potentially other as yet unknown genetic modules. We have demonstrated that the majority of tested toxins can be converted without optimization into effective negative selection systems that vary little in their efficiency and stringency. Furthermore, we have shown that no optimization is needed to use these systems in multiple clinical strains of *E. coli*, including a previously uncharacterized clinical isolate and a well-studied lab strain of *E. coli*, as well as in *S. enterica* Serovar Typhimurium. Therefore, we anticipate that little or no optimization will be needed for the vast majority of *E. coli* strains.

The stringency and generality of our design certainly depends on the availability of tightly regulated promoters in *E. coli* and other enteric bacteria. We have shown that both the rhamnose and arabinose promoters are sufficiently well controlled in single copy to enable bacterial growth when not specifically induced and are very stringent in preventing growth when induced. Based on similar promoter dynamics when present in single copy on the chromosome, we also expect the widely used *lac* promoter to be a viable promoter for driving toxin genes (43,44). Additional promoters native to *E. coli* and other Enterobacteria that respond to pH (45), temperature (46) or oxygenation conditions (47) could also be used. This intentional modularity of transcriptional control, enabling use of different promoters, is particularly valuable for generalization to other bacterial systems.

We noted that, while four of six tested TA system genes could be immediately used in our strategy, *higB* was only viable for negative selection when present on a plasmid in multiple copies, while *yafQ* could not be used for negative selection at all. Interestingly, this implies that the induced expression level from a single copy is insufficient to prohibit growth. In all cases, the 5′ untranslated sequence and ribosome binding sequence were identical among our constructs. Therefore, we suspect that the observed variation in toxicity at the same levels of promoter induction could be due to additional transcriptional (such as pause or termination sites), translational (such as rare codons or mRNA stability), or post-translational effects (such as protein folding or intrinsic toxicity thresholds) unique to individual toxins. Regardless of the mechanism, however, our results imply that toxins differ in their ‘effective toxicity’ when we controlled copy number and promoter induction; this concept of a threshold for toxicity has been introduced previously in relation to levels of the antitoxin within a cell (48). Because the toxins themselves are known to function in a broad range of bacteria (25,26) as well as eukaryotes (yeast) (49), toxin genes that are permissive for cell growth at low transcription levels may enable the use of less optimal (less tight) inducible promoters. Furthermore, due to the large number of toxin-antitoxin systems (and other bacterial toxins from phages and secretion systems) present in public databases from genome sequencing efforts (cataloged, for example, in TADB (50), RASTA (51) and the PanDaTox (52) databases), it seems likely that effective toxicity will vary greatly, especially if codon usage or transcriptional controls within the coding sequences are not removed. Indeed, one could imagine collecting a database of toxin genes that are characterized based on the threshold transcriptional or induction level required for toxicity; this could then be queried to identify reasonable candidates for use with existing inducible promoters to generate additional negative selection systems depending on the available genetic tools in a given system.

We have performed numerous chromosomal manipulations in UTI89 and other lab and clinical *E. coli* strains using all variants of these negative selection systems. The construction of definitive strains for genetic tests in clinical isolates is no longer less convenient than in lab strains. However, we have relied on the widely used Red recombinase system from phage λ. Expression of this system in *E. coli* is known to be mutagenic, and we use it in each of two steps to manipulate the chromosome. While following published recommendations for limiting the length of recombinase expression (22), the possibility still remains that unselected mutations may be induced that could confound interpretation of downstream experiments. To definitively address this possibility, we used full genome sequencing of one of our allelic replacement strains and found no unexpected mutations of any class, including SNPs, small indels, or large scale rearrangements. Furthermore, phenotypic testing of a panel of FimH mutants, all constructed by allelic replacement, showed perfect concordance with previously published results (in which the strains carried a linked positive selection marker).

Our system exceeds the reported stringency of selection (measured using stringency frequencies) of the next best reported system (*tetA–sacB* in MG1655, W3110 and/or DH10B; 6 × 10^−7^ (3)) by up to 60× in lab strains of *E. coli* (kan-P*_rhaB_-tse2* in MG1655; 1.11×10^−8^ as measured by plating 10^11^ CFU); this begins to approach (within 5×) the selection stringency frequency of positively selectable antibiotic markers in some strains we have tested (kanamycin in *E. coli* EDL933; 2.40 × 10^−9^). The stringency frequency in clinical strains (kan-P*_rhaB_-relE* in UTI89; 3.31 × 10^−8^) is 17-fold better than the *tetA–sacB* system as measured in lab strains, and it remains high without the need for strain- or species-specific optimization of transformation or growth conditions. We have further measured mutation *rates* for growth on restrictive conditions and find that these also correspond well with expected minimum theoretical rates for inactivating mutations as calculated by previously reported genomic mutation rates in *E. coli* (34,35). We have used the more easily measured stringency frequencies to compare the different systems. This is similar to previous screens of mutation rates in *E. coli* (31–33), though perhaps not fully rigorous. We note, however, that our stringency frequencies indicate that kanamycin is more stringent than our negative selection system (namely, using P*_rhaB_-tse2*), and we obtain the same result when making a more careful measurement of mutation rate. Furthermore, measurement of mutation frequencies is able to detect mutator strains as those with higher mutation frequencies (32,33). Thus our and previous data both argue that measurement of stringency (mutation) frequencies is reasonable for a relative comparison of mutation rates, a notion further supported by additional theoretical analysis of fluctuation tests for mutation rates (53).

Although mutation rates and frequencies are not directly comparable, we note here two further observations inspired by a discussion with one of the reviewers. First, our expected minimum mutation rate (0.9 × 10^−8^) and calculated mutation rate (2.45 × 10^−8^) are both similar to the mutation rates towards resistance to phage measured by Luria and Delbruck themselves (1.1 × 10^−8^–4.1 × 10^−8^) (17). In looking closely at their original data, the culture sizes (0.2–10 ml with 1 × 10^8^–4×10^10^ CFU in each) are also similar to ours. The ‘average’ number of resistant bacteria measured in each of their cultures, due to the jackpot effect, is higher than the number of resistant bacteria measured in the majority of their cultures (50–95% of cultures in each experiment have fewer resistant bacteria than the average), which forms part of the motivation for developing the fluctuation test instead of relying on mutation frequencies. Furthermore, using a ‘simple expectation’ calculation of (# of bacteria) × (mutation rate) as an arbitrary cutoff, Luria and Delbruck observe an average frequency of mutant CFU above this number, while we see frequencies of background growth near or below this number. This again highlights the distinction between mutation frequencies and rates, arguing that direct measurements of rates are more reliable. This observation may also indicate that we have not sampled enough independent cultures to capture the high variance in the number of mutations present per culture; unfortunately, due to the way we performed the fluctuation tests, we did not collect data on the number of resistant CFU per culture.

However, as a second point, we do note another interesting set of data that provides an informative comparison with our work. Recently, Luria–Delbruck fluctuation tests were performed on *E. coli* for resistance to rifampicin, in the context of examining the relationship of cell density with mutation rate (54); the measured range of mutation rates to rifampicin resistance spanned ∼2 × 10^−9^ to 1 × 10^−8^. Several earlier studies have examined mutation to rifampicin resistance in *E. coli* using simple frequency tests; two large studies of *E. coli* isolates obtained mutation frequencies of 2.5 × 10^−9^ to 1 × 10^−8^ (31) and 5 × 10^−9^ to 5 × 10^−8^ (32), and a third study of *E. coli* and *Salmonella* obtained <1 × 10^−8^ to 5 × 10^−8^ (33). The mutation rate and frequency studies differ in the strains and media used, but overall the results match our data in that the (more simply measured) mutation frequencies are quantitatively similar to the mutation rates measured using the fluctuation tests. Furthermore, we also note a quantitative similarity between the stringency frequency (2.30 × 10^−10^) and mutation rate (1.11 × 10^−10^) that we measured for kanamycin resistance. Again, we note only that the quantitative similarity between stringency (mutation) *frequencies* and mutation *rates* that we observe in our data is mirrored in published data and present in multiple experiments with different selection cassettes; we therefore suggest that this likely indicates our measurements are accurate. However, the conceptual separation of rates and frequencies must still be maintained. For most other negative selection cassettes, however, only stringency frequencies are available, and the following discussion of stringencies should be considered in the context of the foregoing discussion.

Unlike several commonly used systems including *sacB* or *tetA*, our negative selection cassettes appear to come very close to the theoretical maximal stringency (based on mutation rate) for genes of their size, which may account for their superiority over these systems. Although the theoretical maximal stringency for *sacB* is between 1.67 × 10^−8^ and 4.25 × 10^−7^, the reported stringencies (usually using stringency frequency) measured for this system are much lower (*E*. coli HS996; 2.3 × 10^−5^ (20)). Similarly, although the theoretical maximal stringency for *tetA* is between 1.33 × 10^−8^ and 3.40 × 10^−7^, the reported stringencies in different host strains and at different nickel concentrations vary between 10^−4^ and 10^−6^ (1). These systems fall short of expectations in their behavior by 100- to 1000-fold (assuming the correspondence between mutation frequencies and rates should also hold for these systems). Why is our system more robust to selection conditions and changing host strains than previous systems? We speculate that several aspects of our design contribute to higher stringency. First, we are using toxins that have presumably evolved for the purpose of efficiently stopping cell growth; in contrast, tetracycline resistance and the *sacB* gene were evolved for other purposes, and their use for negative selection is based on leveraging other properties they confer on cells. Second, we have included several features that enable us to have ‘tight’ control over toxin induction, including transcriptional terminators and the well characterized rhamnose and arabinose promoters to reduce basal uninduced expression; and the use of the native *rhaB* RBS sequence and minimal media with a single carbon source to ensure good ‘induced’ expression. The combination of an inducible toxin, therefore, for arresting cell growth becomes reliably stringent in different strains and species so long as we can maintain low or no basal expression. A further potential improvement may also be gained by using genomic templates for amplification of the positive–negative selection cassette, thereby eliminating the possibility of the template plasmids conferring resistance during recombination, despite their reliance on conditional replication origins. In contrast, the variable selection mediated by *tetA* or *sacB* may be due to their overall expression levels in and membrane properties of different strains. We finally note that generality and high stringency is common for negative selection systems that require special host genotypes (such as *tolC* (41)*, thyA* (6) and *neo-rpsl* (7)); we have now provided this benefit to unmodified lab and clinical isolates of *E. coli* and *Salmonella* and potentially other Enterobacteriaceae.

In practice, stringency does seem to vary for our negative selection system in different experimental settings, but no more than it does for positive selection markers. For example, in the reimplementation of P1 transduction using negative selection with MG1655 carrying the *tse2* toxin, we saw a higher number of background colonies (200 out of 10^9^ CFU, giving a background frequency of 5 × 10^−6^). However, we have observed this result with positive selection markers as well. Kanamycin stringency frequency is 2.3 × 10^−10^, but use of the Datsenko and Wanner protocol with Red recombinase leads to background colonies (not the desired recombinants and not able to regrow on kanamycin upon passage) growing on kanamycin plates at a frequency higher than this. This discrepancy in kanamycin stringency frequency may be due to the mutagenic effect of Red recombinase expression, although we and others (22) see no evidence for higher mutation when Red recombinase expression is limited in time. Similarly, we have no good explanation for the higher background rate when performing P1 transduction compared to simple titering on rhamnose plates. However, the variation in stringency seems to apply to both negative and positive selection markers.

The generally high stringency of our negative selection system is sufficient to enable previously inaccessible large scale genomic manipulations. We have carried out an initial proof of concept experiment which shows that the system is powerful enough to select for clones of UTI89 that have been transformed with the correct 10 kb fragment of the CFT073 genome (0.2% of the total genome) and then recombined it into the correct *ompC* locus. Potentially, additional development of this technology to reduce background colonies and improve genomic DNA transfer length and efficiency, such as by using Hfr strains for conjugation or transducing lysates, would enable the transfer of entire swaths of genomic DNA from unrelated strains, subject to flanking homology. Combined with transposon engineering, a pool of strains containing randomly inserted negative selectable markers could then be used to create libraries of wholesale allelic exchange mutants in a procedure we term ‘mass allelic exchange’ (Figure 3C). This would finally enable a practical genetic technology for assigning phenotypic consequences to intra-species polymorphisms as well as close inter-species differences. This enabling of sexual genetics in non-naturally competent bacteria could be especially powerful for dissecting the differences between strains of *E. coli* (or other bacteria) that cause different diseases in humans, a longstanding and perplexing problem.

One drawback of *relE* and several of the other toxins used is that they tend to be bacteriostatic instead of bacteriocidal. This necessitates an extra step of colony purification during standard cloning procedures and must be accounted for in large-scale allelic exchange experiments. However, switching to a bacteriocidal toxin should alleviate this drawback and increase the efficiency and convenience of this system, while possibly simultaneously further broadening the host range.

In summary, we have designed a modular and general negative selection system that is both extremely powerful and highly versatile, potentially enabling generalization to other bacteria. The high selection stringency enables novel and improved applications including generalized allelic exchange between arbitrary *E. coli* strains (or even between different species). This negative selection module should therefore be particularly useful in the dissection of bacterial virulence as well as regulation, physiology, speciation, and evolution. With further optimization, libraries of strain hybrids could be created that would surmount drawbacks inherent to positive selection based hybrids (55), enabling sexual genetic strategies to systematically evaluate the thousands of DNA polymorphisms that typically separate unrelated clinical isolates of *E. coli* and other bacteria that are not naturally competent.

## SUPPLEMENTARY DATA

Supplementary Data are available at NAR Online.

SUPPLEMENTARY DATA
