# Supplementary material for: A set of powerful negative selection systems for unmodified Enterobacteriaceae
Source: Nucleic Acids Res. 2015 Mar 23;43(13):e83. doi: 10.1093/nar/gkv248 (PMC4513841; doi:10.1093/nar/gkv248)
Supplement: SUPPLEMENTARY DATA [file supp_43_13_e83__index.html]

A set of powerful negative selection systems for unmodified Enterobacteriaceae — A set of powerful negative selection systems for unmodified Enterobacteriaceae — SUPPLEMENTARY DATA 

# A set of powerful negative selection systems for unmodified Enterobacteriaceae

## SUPPLEMENTARY DATA

**Files in this Data Supplement:**

- SUPPLEMENTARY DATA
