# Supplementary material for: A set of powerful negative selection systems for unmodified Enterobacteriaceae
Source: Nucleic Acids Res. 2015 Mar 23;43(13):e83. doi: 10.1093/nar/gkv248 (PMC4513841; doi:10.1093/nar/gkv248)

Supplemental Figure legends

Supplementary Figure 1. Counter-selection using *tetA*-dependent Ni^{2+} toxicity in UTI89. Bars represent CFU/ml. Data represents the mean CFU/ml of three biological replicates. ND, not determined. LOD, limit of detection. Strains are indicated on the X-axis. pBR322 (copy number ~20) and pACYC184 (copy number ~15) confer tetracycline resistance with the *tetA* gene. UTI89::tet has the *tetA* gene integrated in single copy on the chromosome at the HK locus. At a Ni^{2+} concentration of 3mM, there is a 10^4-fold difference in CFU between the wild type UTI89 (tetracycline sensitive) and UTI89/pBR322.

Supplementary Figure 2. Heat map of BLAST analysis of type II toxin genes in different *E.coli* genomes. Each row represents the presence (red) or absence (beige) of a toxin gene (indicated on the right) in each of 55 complete non K-12 *E. coli* genomes (indicated on the bottom). Presence is defined as alignment over 80% of the gene sequence by the blastn program (see methods).

Supplementary Figure 3. Verification of *ompC* knockout and replacement in UTI89. (A) PCR confirmation of knockout and complementation at the *ompC* locus. Lane 1, 10kb marker. Lanes 2-4, PCR products from indicated strains using primer pair P21/P22. A 1.3kb band is expected for the wt locus, and a 3kb band is expected for the knockout. Lanes 5-7, PCR products from indicated strains using primer pair P3/P22. A 900bp band indicates integration of the kan-P_{rhaB}-*relE* at the targeted (*ompC*) locus. (B) Phenotypic confirmation of *ompC* knockout and complement. Wt UTI89 ($kan^S rha^R$), SLC-516 (*ompC*::kan-P_{rhaB}-*relE*; $kan^R rha^S$), and SLC-517 (*ompC*::$ompC^{UTI89}$; $kan^S rha^R$) were streaked out on LB-kanamycin and M9-rhamnose plates to confirm their phenotype. (C) Immunoblot detection of OmpC protein. Primary antibodies were 4H15 (monoclonal to OmpC loop 7, left blot) and 4K3 (monoclonal to OmpC loop 5, right blot). Wt UTI89 (SLC-6), *ΔompC* control (SLC-17), *ompC*::kan-P_{rhaB}-*relE* knockout (SLC-516), wt *ompC* complement (SLC-517), and L5 OmpC mutant (SLC-559) strains were subjected to western blot analysis. The 4H15 Ab cross-reacts to one non-specific band (indicated by *). Arrows indicate the location of the OmpC protein band in each blot. The same blots were probed with an anti-GroEL antibody as an internal/loading control.

Supplementary Figure 4. Selection stringency tests for *higB* and holin. Data shows the negative selection stringency (CFU/ml on restrictive agar divided by CFU/ml on non-restrictive agar) for *higB* and holin toxic genes. pSLC-240 (pKD4 with P_{rhaB}-*higB*) was propagated in *E. coli* BW23474 at high copy number (250 copies/cell) and kan-P_{rhaB}-*higB* was integrated into the UTI89 chromosome at the *ompC* locus to check stringency when present in a single copy. pSLC-247 (pAH150 with holin) was propagated in *E. coli* BW23474 at high copy number (250 copies/cell) and kan-P_{araB}-holin was integrated into the CFT073 chromosome at the *ompC* locus. Data represents the average of the log-transformed values of selection stringencies with error bars indicating standard deviation of the log-transformed stringency values calculated from three independent biological replicates.

Supplementary Figure 5. Positive selection stringency tests for wild type strains followed by strains in which the selection cassette has been replaced with the wild type or a different allele in a subsequent negative selection step. A) Stringency for replacement of the dual selection cassette in different loci.

Data shows the selection stringency for wild type strains (on the left) followed by selection stringency for strains in which the selection cassette has been replaced with the wild type allele in a subsequent negative selection step (on the right). (**B**) Stringency for replacement of the dual selection cassette in different strains of *E. coli* and in *Salmonella*. Data shows the selection stringency for each wild type strains (on the left) followed by selection stringency for strains in which the selection cassette has been replaced with a different allele from UTI89 in a subsequent negative selection step (on the right). For graphs in **(A and B)**, data represents the average of the log-transformed values of selection stringencies with error bars indicating standard deviation of the log-transformed stringency values calculated from three independent biological replicates.

Supplementary Figure 6. Verification of stringencies and mutation rates. Stringency tests for SLC-568 (MG1655 *hsdS*::*neo* P_{rhaB} *tse*2) as well as SLC-657 (re-constructed MG1655 *hsdS*::*neo* P_{rhaB} *tse*2). Data shows the negative selection stringency for SLC-568 with two different inocula (10^{10}CFU and 10^{11} CFU) and SLC-657. Data represents the average of the log-transformed values of selection stringencies with error bars indicating standard deviation of the log-transformed stringency values calculated from three independent biological replicates.

Supplementary Tables

Supplementary Table 1: Strains and plasmids used in the study.

Strains	Genotype and/or relevant characteristics	Source/reference
BW23474		Haldimann et al.,1997
BW23473		Haldimann et al.,1997
UTI89	Clinical isolate	Chen et al., 2009
SLC-17	UTI89 *ompC*::kan	This study
SLC-551	UTI89 HK2K-p2-tet	This study
SLC-620	UTI89 *ompC*::*cat-sacB*	This study
SLC-516	UTI89 *ompC*::*neo*-P_{rhaB}-*relE*	This study
SLC-517	UTI89 *ompC*^{UTI89}	This study
SLC-521	UTI89 *ompC*::*cat*-P_{rhaB}-*relE*	This study
SLC-519	UTI89 *ompC*::*neo*-P_{araB}-*relE*	This study
SLC-523	UTI89 *ompC*::*neo*-P_{rhaB}-*mqsR*	This study
SLC-525	UTI89 *ompC*::*neo*-P_{rhaB}-*chpB*	This study
SLC-527	UTI89 *ompC*::*neo*-P_{rhaB}-*yhaV*	This study
SLC-529	UTI89 *ompC*::*neo*-P_{rhaB}- *higB*	This study
SLC-538	UTI89 *ompC*^{UTI89}	This study
SLC-539	UTI89 *ompC* ^{UTI89}	This study
SLC-540	UTI89 *ompC* ^{UTI89}	This study
SLC-541	UTI89 *ompC* ^{UTI89}	This study

SLC-502	UTI89 *fimH*::*neo*-P_{rhaB}-*relE*	This study
SLC-463	UTI89 *fimH*^{UTI89}	This study
SLC-464	UTI89 *fimH*-A62S	This study
SLC-465	UTI89 *fimH*-A27V-V163A	This study
SLC-478	UTI89 *fimH*-Q133K	This study
SLC-560	UTI89 *fimH::FRT mlaA*::*neo*-P_{rhaB}-*relE*	This study
SLC-561	UTI89 *fimH::FRT mlaA*::*mlaA*^{UTI89}	This study
SLC-565	UTI89 *hsdS*::*neo*-P_{rhaB}-*relE*	This study
SLC-567	UTI89 *hsdS*^{K12}	This study
SLC-557	UTI89 *ompC*::*neo*-P_{rhaB}-*relE*	This study
SLC-559	UTI89 *ompC* Loop 5 SCAN mutant	This study
SLC-562	EDL933 *ompC*^{UTI89}	This study
SLC-564	14028S *ompC*^{UTI89}	This study
SLC-563	CFT073 *ompC*^{UTI89}	This study
SLC-578	UTI89 *ompC*^{CFT073}	This study
SLC-579	UTI89 *ompC*^{CFT073}	This study
Salmonella Typhimurium		
14028S		Jarvik et al., 2010
SLC-530	14028S *ompC*::*neo*-P_{rhaB}-*mqsR*	This study
SLC-534	14028S *ompC* ^{UTI89}	This study
E.coli CFT073	Clinical isolate	Welch et al., 2002
SLC-535	CFT073 *ompC*::*neo*-P_{rhaB}-*relE*	This study
SLC-537	CFT073 *ompC* ^{UTI89}	This study
SLC-644	CFT073 *ompC*::*neo*-P_{rhaB}-*tse2*	This study
SLC-645	CFT073 *ompC* ^{UTI89}	This study
E.coli MG1655		
SLC-568	MG1655 *hsdS*::*neo*-P_{rhaB}-*tse*2	This study
SLC-570	MG1655 *hsd*S^{UTI89}	This study
SLC-657	MG1655 *hsdS*::*neo*-P_{rhaB}-*tse*2	This study
SLC-658	MG1655 *hsdS*::*neo*-P_{rhaB}	This study
E.coli EDL933		Riley et al., 1983
SLC-575	EDL933 *ompC*::*neo*-P_{rhaB}-*relE*	This study
SLC-562	EDL933 *ompC* ^{UTI89}	This study
SLC-642	EDL933 *ompC*::*neo*-P_{rhaB}-*tse2*	This study
SLC-643	EDL933 *ompC* ^{UTI89}	This study
TOP2515	Clinical isolate	Chen et al., 2013

SLC-646	TOP2515 *ompC*::*neo*-P$_{rhaB}$-*tse2*	This study
SLC-647	TOP2515 *ompC* ^{UTI89}	This study
Plasmids	Genotype and/or relevant characteristics	Source/reference
pKD3		Datsenko and Wanner, 2000
pKD4		Datsenko and Wanner, 2000
pAH120		Haldimann and Wanner, 2001
pAH150		Haldimann and Wanner, 2001
pSLC-243	SmaI, AatII, SacII *cat*	This study
pSLC-217	*neo* P$_{rhaB}$ *relE*	This study
pSLC-242	*cat* P$_{rhaB}$ *relE*	This study
pSLC-237	*neo* P$_{araB}$ *relE*	This study
pSLC-238	*neo* P$_{rhaB}$ *chpB*	This study
pSLC-239	*neo* P$_{rhaB}$ *yhaV*	This study
pSLC-240	*neo* P$_{rhaB}$ *higB*	This study
pSLC-241	*neo* P$_{rhaB}$ *mqsR*	This study
pSLC-248	*neo* P$_{rhaB}$ holin	This study
pSLC-247	pAH150-holin	This study
pSLC-246	*neo* P$_{rhaB}$ *tse2*	This study
pSLC-163	pAH120-*yafQ*	This study
pACYC184		Chang and Cohen, 1978
pBR322		Sutcliffe, 1979
pKD46		Murphy and Campellone, 2003
pKM208		Murphy and Campellone, 2003
pEL04		National Cancer Institute

Supplementary Table 2: Toxin genes identified.

Systematic name	Toxin gene	TA system	Toxin length (aa)	Present in UTI89	# of non-K12 genomes	% of non-K12 genomes
b0225	*yafQ*	dinJ/yafQ	92	No	33	60.0%
b0233	*yafO*	yafN/yafO	132	Yes	28	50.9%
b0245	*ykfI*	yafW/ykfI	113	No	1	1.8%
b1357	*ydaS*	ydaS/ydaT	98	No	7	12.7%
b1507	*hipA*	hipB/hipA	440	Yes	54	98.2%
b1563	*relE*	relB/relE	95	No	12	21.8%
b2005	*cbtA*	yeeU/cbtA	124	Yes	44	80.0%
b2619	*ratA*	ratA/yfjF	158	Yes	55	100.0%
b2630	*rnlA*	rnlA/rnlB	357	No	0	0.0%
b2646	*ypjF*	yfjZ/ypjF	109	No	0	0.0%
b2782	*mazF*	mazE/mazF	111	Yes	46	83.6%
b2896	*cptA*	cptA/cptB	135	Yes	55	100.0%
b3022	*mqsR*	mqsR/mqsA	98	No	13	23.6%
b3083	*higB*	higB/higA	104	No	46	83.6%
b3130	*yhaV*	prlF/yhaV	154	No	48	87.3%
b4225	*chipBK*	chpBI/chpBK	116	No	30	54.5%
b4517	*gnsA*	gnsA/ymcE	57	Yes	55	100.0%
b4532	*hicA*	hicA/hicB	58	No	35	63.6%
b4539	*yoeB*	yefM/yoeB	84	Yes	40	72.7%
b4566	*yjhX*	yjhX/yjhQ	85	No	11	20.0%

Supplementary Table 3: Different counter-selection cassettes tested at different loci in UTI89 as well as other organisms.

Selection cassette	Copy number	Size of dual selection cassette (Kb)	Loci	Organism
neo P_{rhaB} *relE*	1	2.9	*ompC, mlaA, fimH, hsdS*	UTI89, CFT073, EDL933
cat P_{rhaB} *relE*	1	2.4	*ompC*	UTI89
neo P_{araB} *relE*	1	2.9	*ompC*	UTI89
neo P_{rhaB} *chpB*	1	2.9	*ompC*	UTI89
neo P_{rhaB} *yhaV*	1	3.0	*ompC*	UTI89
neo P_{rhaB} *higB*	250 (pAH120, *pir116*)		*ompC*	UTI89
neo P_{rhaB} *mqsR*	1	2.8	*ompC*	UTI89, *S. typhimurium* 14028S
neo P_{rhaB} *tse*2	1	3.0	*ompC, hsdS*	CFT073, MG1655
neo P_{rhaB} holin	250 (pAH120, *pir116*)		plasmid	BW23474
neo P_{araB} holin	250 (pAH120, *pir116*)		plasmid	BW23474
neo P_{rhaB} *yafQ*	15 (pAH120, *pir*)		plasmid	BW23473

Supplementary Table 4: SDS-EDTA test for *mlaA* strains.

Strain	Genotype	EDTA concentration at which inhibition was observed
E.coli UTI89		2.0mM
SLC-560	UTI89 *fimH::FRT mlaA*::*neo*-P_{rhaB}-*relE*	1.25mM
SLC-561	UTI89 *fimH::FRT mlaA*::*mlaA*^{UTI89}	2.0mM

Supplementary Table 5: HA titres for *fimH* strains

Strain	Genotype	HA titres	
		Using positive selection (residual kanamycin marker)	Using negative selection (no residual marker)
SLC-6	*E.coli* UTI89	8	8
SLC-463	UTI89 *fimH*^{UTI89} complement	8	8
SLC-464	UTI89 *fimH*-A62S	5	5
SLC-465	UTI89 *fimH*-A27V-V163A	7	7
SLC-478	UTI89 *fimH*-Q133K	4	4

Supplementary Table 6: Negative selection stringencies for all tested cassettes

Strains	Negative selection stringency
UTI89 *ompC*::*neo* P_{rhaB} *relE*	3.31×10^{-8}
UTI89 *ompC*::*neo* P_{araB} *relE*	2.00×10^{-7}
UTI89 *ompC*::*cat* P_{rhaB} *relE*	7.14×10^{-8}
UTI89 *ompC*::*neo* P_{rhaB} *mqsR*	1.02×10^{-7}
UTI89 *ompC*::*neo* P_{rhaB} *chpB*	2.41×10^{-7}
UTI89 *ompC*::*neo* P_{rhaB} *yhaV*	1.29×10^{-7}
UTI89 *fimH*::*neo* P_{rhaB} *relE*	8.05×10^{-8}
UTI89 *mlaA*::*neo* P_{rhaB} *relE*	9.13×10^{-8}
UTI89 *hsdS*::*neo* P_{rhaB} *relE*	6.71×10^{-8}
CFT073 *ompC*::*neo* P_{rhaB} *relE*	5.50×10^{-8}
CFT073 *ompC*::*neo* P_{rhaB} *tse2*	5.37×10^{-8}
EDL933 *ompC*::*neo* P_{rhaB} *relE*	9.93×10^{-8}
EDL933 *ompC*::*neo* P_{rhaB} *tse2*	1.08×10^{-7}
S.typhimurium *ompC*::*neo* P_{rhaB} *mqsR*	1.36×10^{-7}
MG1655 *hsdS*::*neo* P_{rhaB} *tse2*	1.96×10^{-8}
MG1655 *hsdS*::*neo* P_{rhaB} *tse2* (as measured by plating 10^{11} CFU)	1.11×10^{-8}
TOP2515 *ompC*::*neo* P_{rhaB} *tse2*	3.50×10^{-7}

Supplementary Table 7: Titres for strains used in selection stringency

Strains	CFU/10ml		
	LB	Rhamnose	LB-kanamycin
UTI89 wild type	1.48×10^{10}	1.45×10^{10}	8.67×10^{0}
UTI89 *ompC*::*neo* P_{rhaB} *relE*	1.27×10^{10}	4.53×10^{2}	1.41×10^{10}
UTI89 *ompC*::ompC^{UTI89}	1.65×10^{10}	1.70×10^{10}	1.41×10^{2}
CFT073 wild type	6.92×10^{9}	7.88×10^{9}	6.67×10^{0}
CFT073 *ompC*::*neo* P_{rhaB} *relE*	6.27×10^{9}	2.99×10^{2}	5.13×10^{9}
CFT073 *ompC*::*ompC*^{UTI89}	5.63×10^{9}	6.20×10^{9}	4.33×10^{0}
EDL933 wild type	6.80×10^{9}	7.44×10^{9}	1.70×10
EDL933 *ompC*::*neo* P_{rhaB} *relE*	5.85×10^{9}	5.29×10^{2}	4.60×10^{9}
EDL933 *ompC*:: *ompC*^{UTI89}	5.76×10^{9}	5.21×10^{9}	1.00×10^{0}
S.typhimurium wild type	8.12×10^{9}	6.33×10^{9}	1.00×10^{0}
S.typhimurium *ompC*::*neo* P_{rhaB} *mqsR*	6.87×10^{9}	9.10×10^{2}	7.13×10^{9}
S.typhimurium *ompC*:: *ompC*^{UTI89}	7.41×10^{9}	7.88×10^{9}	1.00×10^{0}
MG1655 wild type	7.88×10^{9}	5.49×10^{9}	1.00×10^{0}
MG1655 *hsdS*::*neo* P_{rhaB} *tse2*	3.96×10^{9}	4.73×10^{1}	3.33×10^{9}
MG1655 *hsdS*::*hsdS*^{UTI89}	6.83×10^{9}	7.45×10^{9}	2.33×10^{0}
MG1655 *hsdS*::*neo* P_{rhaB} (SLC-658)	2.43×10^{9}	2.60×10^{9}	1.47×10^{9}

Supplementary Table 8: Primers used in this study

No.	Sequence	Notes
1	TGTGTAGGCTGGAGCTGCTTC	pKD4 universal primers
2	CATATGAATATCCTCCTTAG	
3	TAGGCCCATATGGCGTATTTTCTGGAT	Amplification of *relE* from MG1655
4	TAGGCCGGATCCTCAGAGAATGCGTTT	
5	TAGGCCCATATGGAAAAACGCACACCA	Amplification of *mqsR* MG1655
6	TAGGCCGGATCCTTACTTCTCCTTAAA	
7	TAGGCCCATATGGTAAAGAAAAGTGAA	Amplification of *chpB* MG1655
8	TAGGCCGGATCCTTATTCCACCACCGC	
9	TAGGCCCATATGGATTTTCCACAAAGG	Amplification of *yhaV* MG1655
10	TAGGCCGGATCCTCAATGGGTTTCTTC	
11	TAGGCCCATATGCACCTGATAACTCAA	Amplification of *higB* MG1655
12	TAGGCCGGATCCTCATTTTTTCCCCTT	
13	AAAGTGCCACCTGCATCGAT	pAH120 test primers
14	ATCCAGTGCAAAGCTAGC	
15	GATCCTCATATGAGGAGGTAAAATAGATGTCCTACGACTACGAGAAA	Amplification of promoter-toxin module along with terminators from pAH120 and pAH150
16	GATCCTGGATCCCTAGAGCCCGCGGGCTTTTT	
17	GATCCTGGATCCTCATTCGTCCTTCTCCTTCA	Amplification of *ompC*^{UTI89}
18	GATCCTCATATGAGCACCATTCAAACAGG	
19	GGTCGCAAGAGTACACCAAAAAACTGTGTTTGTACGCTGAAAACAATGAAGTGTAGGCTGGAGCTGCTTC	Amplification of positive-negative selection cassette with *ompC*^{UTI89} homology
20	GTTAATAACATGAAAGTTAAAGTACTGTCCCTCCTGGTCCCAGCTCTGCTCATATGAATATCCTCCTTAG	
21	TTTGGCGGGTTGTGGTTTTT	*ompC*^{UTI89} test primers
22	TGCATTTTTGGGGAGAATGG	
23	GGTCGCAAGAGTACACCAAAAAACTGTGTTTGTACGCTGAAAACAATGAA	Amplification of phage holin from UTI89
24	GTTAATAACATGAAAGTTAAAGTACTGTCCCTCCTGGTCCCAGCTCTGCTC	
25	TTAGAACTGGTAAACCAGACCCAGCGCTACGATGTCGTCGGTGTTGATGC	Amplification of *ompC Salmonella*
26	ATGAAAGTTAAAGTACTGTCCCTCCTGGTACCAGCTCTGCTGGTGGCGGG	
27	ATTCGAATGGACGCAAGCGT	*ompC Salmonella* test primers
28	GCGGAGAATGGACTTGCCGA	
29	TTAGAACTGGTAAACCAGACCCAGCGCTACGATGTCGTCGGTGTTGATGCGTGTAGGCTGGAGCTGCTTC	Amplification of positive-negative selection cassette with *ompC Salmonella* homology
30	ATGAAAGTTAAAGTACTGTCCCTCCTGGTACCAGCTC	

	TGCTGGTGGCGGGCATATGAATATCCTCCTTAG	
31	TTAGAACTGGTAAACCAGACCCAGAGCTACGATGTTATCAGTGTTGATGCTGTGTAGGCTGGAGCTGCTTC	Amplification of positive-negative selection cassette with *ompC*^{EDL933} homology
32	ATGAAAGTTAAAGTACTGTCCCTCCTGGTCCCAGCTCTGCTGGTAGCAGGCATATGAATATCCTCCTTAG	
33	TTAGAACTGGTAAACCAGACCCAGAGCTACGATGTTATCAGTGTTGATGC	Amplification of *ompC*^{EDL933}
34	ATGAAAGTTAAAGTACTGTCCCTCCTGGTCCCAGCTCTGCTGGTAGCAGG	
35	CTCATGCGAACGGTCGCAAG	*ompC*^{EDL933} test primers
36	GGGGAGAATGGACTTGCCGA	
37	AGGTAGCACCAACATCAACATATTTCAGGATATCTTCGTCGTCGTAGTTAGTGTAGGCTGGAGCTGCTTC	Amplification of positive-negative selection cassette with *ompC*^{CFT073} homology
38	ATGGCGACCAGACCTACATGCGTCTTGGCTTCAAAGGTGAAACTCAGGTTCATATGAATATCCTCCTTAG	
39	CGCAGGCCCTTTGTTCGATA	*ompC*^{CFT073} test primers
40	CTCCTGGTCCCAGCTCTGCT	
41	AGGTAGCACCAACATCAACATATTTCAGGATATCTTCGTCGTCGTAGTTA	Amplification of *ompC*^{CFT073}
42	ATGGCGACCAGACCTACATGCGTCTTGGCTTCAAAGGTGAAACTCAGGTT	
43	GGTCGCAAGAGTACACCAAAAAACTGTGTTTGTACGCTGAAAACAATGAAGCTTGCAGTGGGCTTACATG	Amplification of positive-negative selection cassette
44	GTTAATAACATGAAAGTTAAAGTACTGTCCCTCCTGGTCCCAGCTCTGCTTCAGAGCAGGATCGACGTCC	with *ompC*^{UTI89} homology without FRT sites
45	TCATGACGCTGGAAGTACGCTTCACGCACCATAATATAAGGATCGGAGGAGTGTAGGCTGGAGCTGCTTC	Amplification of positive-negative selection cassette with *mlaA*^{UTI89} homology
46	CGTTCTGACCCGTTAGAAGGGTTTAACCGCACTATGTACAACTTCAACTTCATATGAATATCCTCCTTAG	
47	CGGATTTTCCTGCGGTTTGA	*mlaA*^{UTI89} test primers
48	GGCGCTTGCTCTGGGAACTA	
49	CGTTCTGACCCGTTAGAAGGGTTTAACCGCACTATGTACAACTTCAACTT	Amplification of *mlaA*^{UTI89}
50	TCATGACGCTGGAAGTACGCTTCACGCACCATAATATAAGGATCGGAGGA	
51	AAAAAGCGCACCGGAAAGGTGCGCC	*hsdS* ^{MG1655} test primers
52	CAGGCGCTGTCTGAACTGGA	
53	TCTGCTTCGAACGGTTGTGC	*hsdS* ^{UTI89} test primers
54	CGCCGAAGAGACGGAAATTG	
55	TCAGGACTTTTTACGCGAGGCTTTTTTACCCCCGCTG	Amplification of positive-negative selection

56	GCTGCGCGTTCAGGCTTGCAGTGGGCTTACATG ATGAGTGCTGGGAAATTGCCGGAGGGGTGGGAACAG ATTGAAATAGGCGATCAGAGCAGGATCGACGTCC	cassette with *hsdS*^{UTI89} homology
57	CCGCATCGCGCTAAATACCTGGATATATCATCAGTAA ATACAGGGAAAGTCCAGCTAAAAATAGAATAAAATGG G	Amplification of *hsdS*^{MG1655} complemented into UTI89
58	AGCGATGGGTGAACTGGTACAGGCGCTGTCTGAACT GGATGCGCTGATGCGTGAACTGGGGGCGAGCGATG AGGC	
59	TCAGGATTTTTTACGTGAGGCTTTTTTACCCCCGCTA GCTGCGCGTTCAGCATGAGAGCTTAGTACGTTA	Amplification of positive-negative selection cassette with *hsdS*^{MG1655} homology
60	ATGAGTGCGGGGAAATTGCCGGAGGGGTGGGTTATC GCCCCAGTATCTACATCCAGTGCAAAGCTAGA	
61	GCCGATTTGCAGCGTCAGTTGCTGGAAGAAGCGTTT GGTGGGGTGAAGGAATGAGTGCTGGGAAATTGCCGG AGG	Amplification of *hsdS*^{UTI89} complemented into MG1655
62	GGCGTGAAATAATAAAAAGCGCACCGGAAAGGTGCG CCAGAAAATAATGTTCAGGACTTTTTACGCGAGGCTT TT	
63	GCAGTTTAAACATCTTCGTG	Amplification and sequencing of region around UTI89 *ompC*
64	CGCACATGTTGCTGAAACGT	
65	TCTCGATTGATATCGAACAA	
66	GTTATTAACCCTCTGTTATA	
67	TCGATACCTGGCGTTACGTG	
68	CTCATCATCGCTTAAAAGCA	
69	GCGATCTCCAGCTCCAAACGTACTTCCTCCTCCTCCT CCGCTGCTGCTGCTGGTTCCGGCGACCGTGCTGAAA CCTAC	Overlapping PCR primers to create UTI89 *ompC* mutant
70	GTAGGTTTCAGCACGGTCGCCGGAACCAGCAGCAGC AGCGGAGGAGGAGGAGGAAGTACGTTTGGAGCTGG AGATCGC	

Supplementary Figure 1

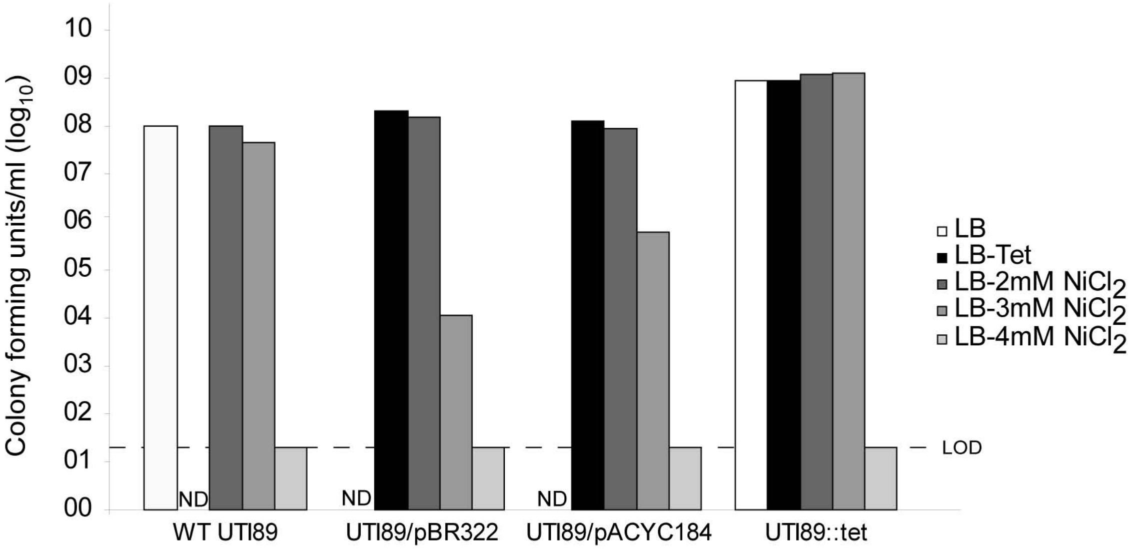

Supplementary Figure 2

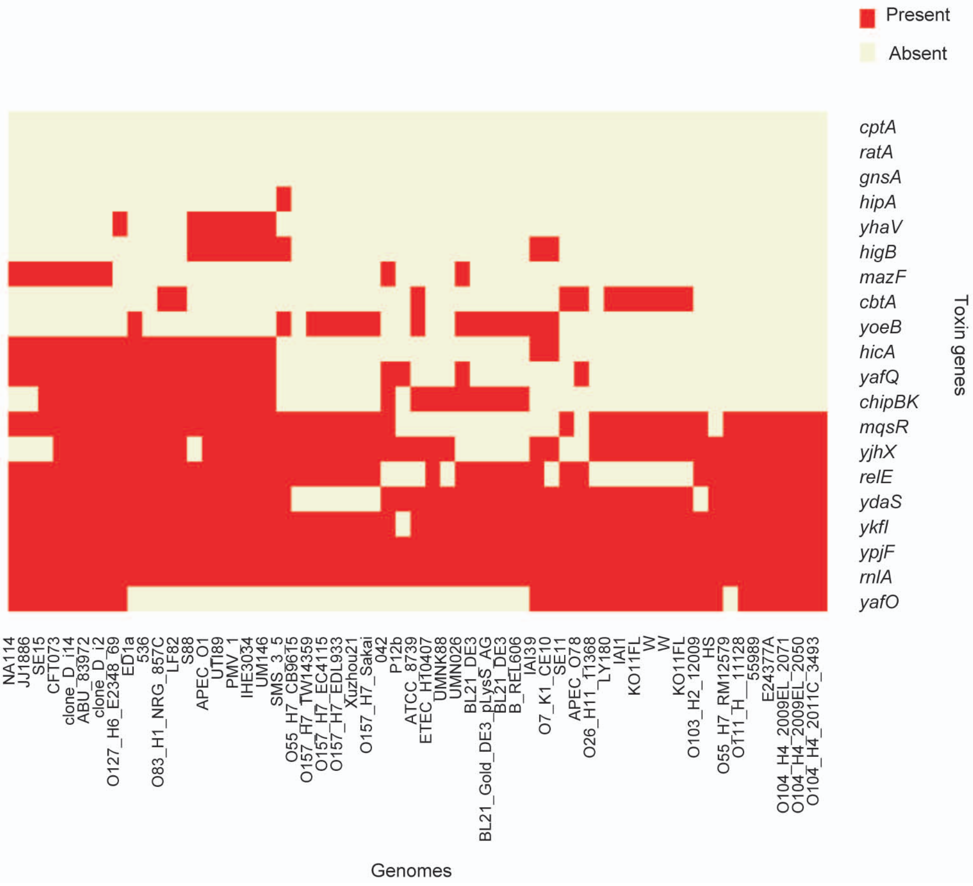

Supplementary Figure 3

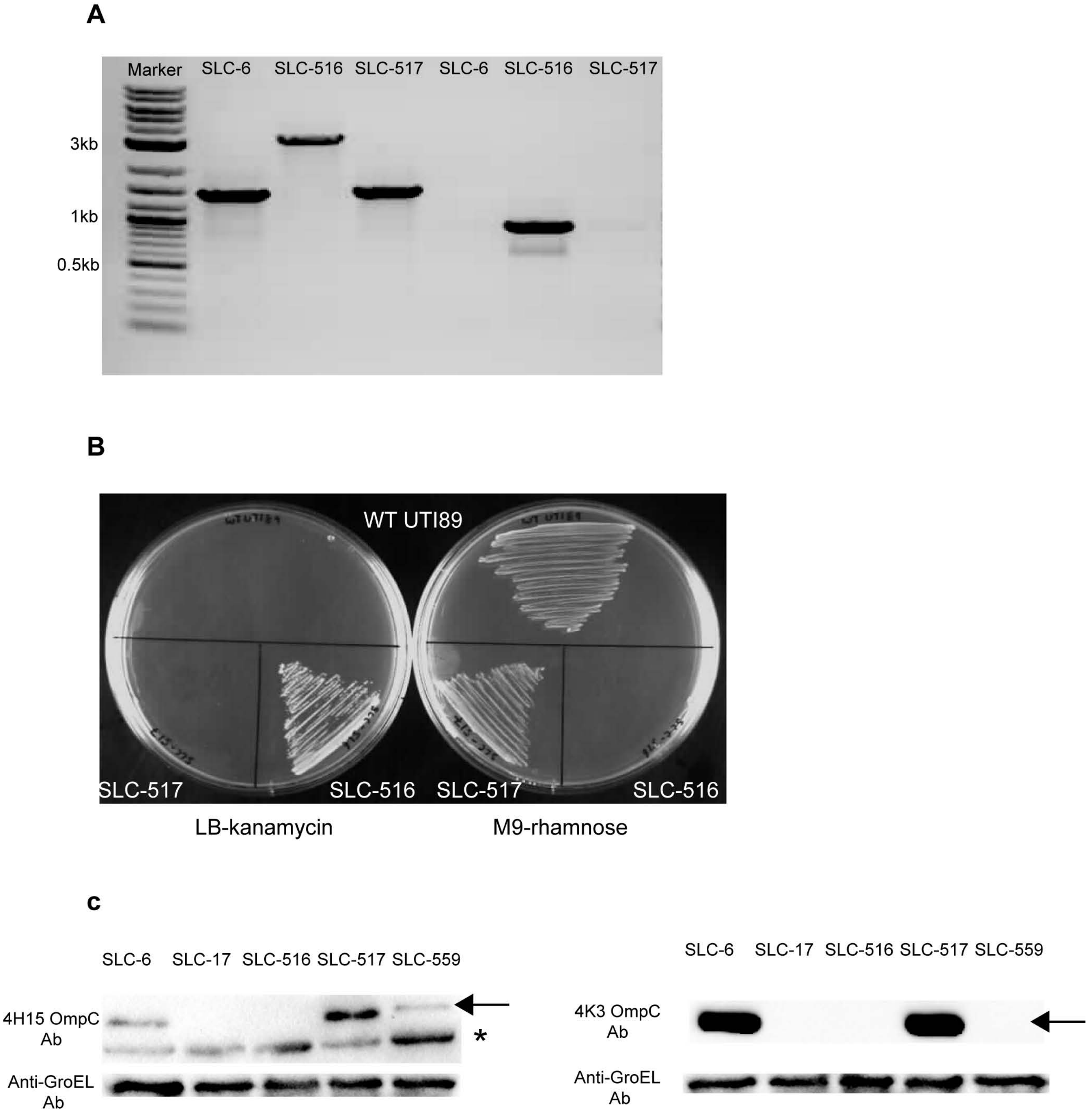

Supplementary Figure 4

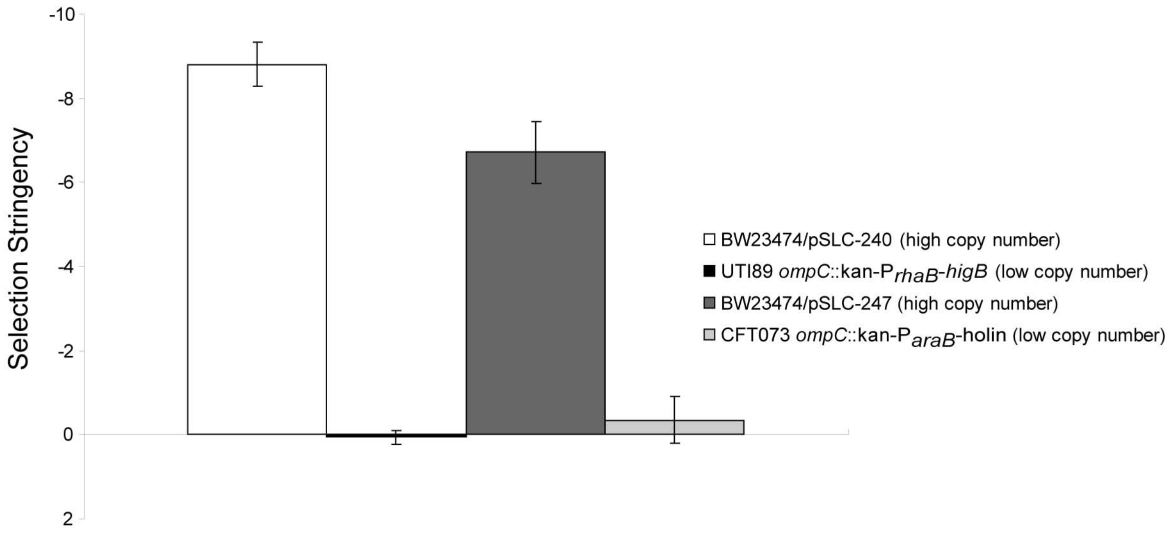

Supplementary Figure 5

A

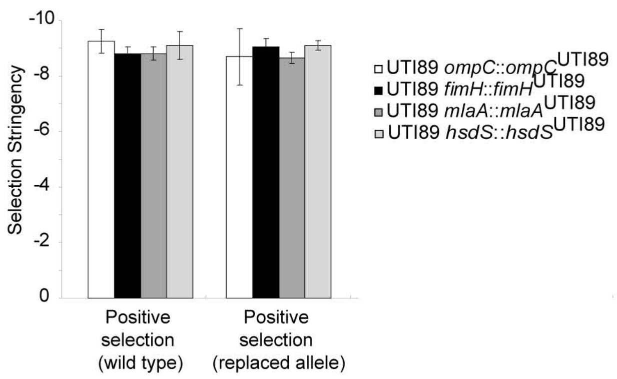

B

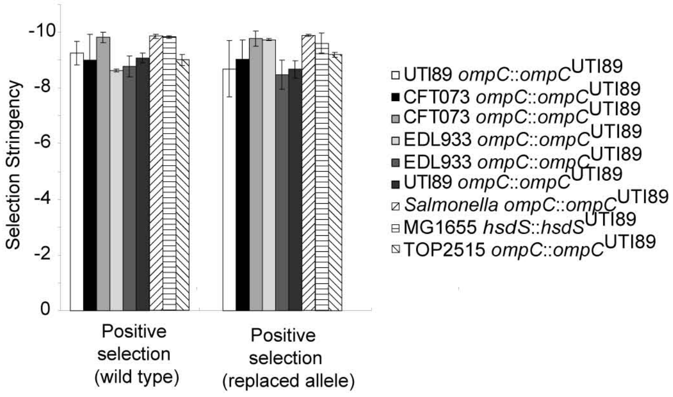

Supplementary Figure 6

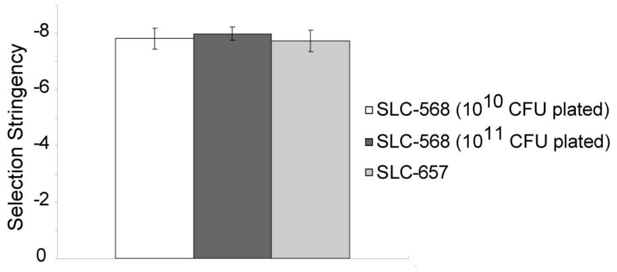

Supplement: SUPPLEMENTARY DATA [file supp_gkv248_nar-02773-met-g-2014-File005.pdf]
